# Processing canonical and non-canonical word orders in Turkish: behavioral and ERP evidence from adults and children

**DOI:** 10.3389/fnhum.2026.1968429

**Published:** 2026-10-07

**Authors:** Medine Dudu, Yağmur Köyel Yıldırım, Talat Bulut

**Affiliations:** 1 Institute of Health Sciences, Istanbul Medipol University, Istanbul, Türkiye; 2 Department of Speech and Language Therapy, Istanbul Medipol University, Istanbul, Türkiye; 3 Vocational School of Health Services, Altınbaş University, Istanbul, Türkiye; 4 Max Planck Institute for Psycholinguistics, Neurobiology of Language Department, Nijmegen, Netherlands

**Keywords:** case marking, event-related potentials (ERPs), language development, morphosyntax, non-canonical word order, sentence comprehension, thematic-role assignment, Turkish

## Abstract

**Introduction:**

Turkish permits grammatical non-canonical word order while morphological case marking disambiguates thematic roles.

**Methods:**

We examined comprehension accuracy and verb-locked event-related potentials (ERPs) to Turkish canonical subject-object-verb (SOV) and non-canonical object-subject-verb (OSV) sentences in adults (*n* = 67; 18–44 years) and children (*n* = 37; 4.3–9.7 years).

**Results:**

Accuracy was lower for OSV than SOV in both groups, with a significantly larger SOV-OSV accuracy difference in children. Adults showed a late posterior OSV > SOV positivity, with significant effects in several prespecified windows within the 600–1,200-ms interval, consistent with increased sentence-final processing demands associated with the non-canonical configuration at the final verb. Children showed no reliable corresponding positivity after correction for multiple comparisons. Critically, however, Group × Sentence Type interactions did not survive correction, providing insufficient evidence that the magnitude of the SOV-OSV ERP effect differed between adults and children. Within children, chronological age showed no reliable linear or quadratic association with the behavioral or ERP SOV-OSV difference across the sampled age range. By contrast, stronger grammatical ability predicted higher comprehension accuracy in both sentence types beyond age; the same general pattern was obtained for broader language ability. Neither grammatical nor broader language ability reliably modulated the SOV-OSV ERP difference.

**Discussion:**

Overall, the findings suggest that the availability of morphosyntactic case cues does not by itself entail adult-like efficiency in integrating those cues during sentence comprehension, and that developmental patterns may be expressed differently across behavioral and electrophysiological measures.

## 1 Introduction

### 1.1 Turkish word order and case marking

Successful sentence comprehension requires listeners to determine how the constituents of a sentence relate to one another and, ultimately, *who did what to whom*. Languages differ, however, in the relative contribution of cues such as constituent order and morphological case marking to this process. Turkish provides a particularly informative case because it combines rich case morphology with relatively flexible constituent order. Although subject–object–verb (SOV) is generally regarded as the canonical order, other permutations are grammatical under appropriate discourse conditions, with case marking helping to preserve the intended grammatical and thematic relations (Erguvanli Taylan, 1984; Göksel and Kerslake, 2005; Kornfilt, 2018), as illustrated in (1). In both sentences, accusative marking identifies the patient despite the change in constituent order. Flexibility of word order does not imply that the different orders are equally frequent or functionally equivalent. In the BOUN Treebank, for example, among clauses in which both main arguments were overt, SOV accounted for 59.53% of the sentences, whereas object–subject–verb (OSV) accounted for only 4.46% (Türk et al., 2022).

**(1)**

a. SOV (Agent-Patient-Action) *Palyaço futbolcu-yu çek-iyor*. clown football.player-ACC pull-PROG.3SG “The clown is pulling the football player.”

b. OSV Word Order (Patient-Agent-Action) *Palyaço-yu futbolcu çek-iyor*. clown-ACC football.player pull-PROG.3SG “The football player is pulling the clown.”

ACC: Accusative, PROG: Progressive, 3SG: Third person singular.

Studies of simple word-order contrasts have shown a processing advantage for canonical SOV over non-canonical OSV sentences in Turkish adults (Ergin and Stromswold, 2023; Bulut and Günhan Senol, 2026). In Bulut and Günhan Senol (2026), for example, canonical SOV sentences were comprehended more accurately and with shorter response latencies than matched reversible OSV sentences and were also read faster at both the first noun phrase and the sentence-final verb. Corpus-level structural frequency, however, does not imply equivalent linguistic experience across speakers. In adult native speakers of English, print exposure and education are associated with individual differences in grammar, vocabulary, and collocational knowledge (Dabrowska, 2018), whereas in adult native speakers of Turkish, print exposure was associated with how speakers extended an inflectional pattern to novel verbs (Gedik, 2024). The processing advantage for canonical SOV is not invariant, however, because the interpretation and processing of non-canonical orders are sensitive to discourse and information-structural context (Işsever, 2003; Kahraman and Hirose, 2018). Constituent order in Turkish is closely related to functions such as topicalization, focus, backgrounding, and information status (Erguvanli Taylan, 1984; Işsever, 2003; Türk et al., 2022). Thus, non-canonical OSV word order provides a useful contrast in which the sentence remains grammatical and its thematic relations can be recovered morphosyntactically, while departing from the dominant canonical order.

Turkish-speaking children acquire and exploit case morphology early in development. Accusative and dative marking emerge early in child Turkish (Ketrez and Aksu-Koç, 2009), and children rely strongly on case information when assigning thematic roles (Slobin and Bever, 1982). Eye-tracking evidence further shows that by 4 years of age, children can use case marking incrementally and predictively during spoken sentence comprehension, before the verb is encountered (Özge et al., 2019). More recent visual-world evidence likewise indicates that Turkish-speaking children and adults use overt accusative marking online in non-canonical sentences, with effects emerging shortly after the relevant case-marked noun phrase, whereas only adults showed sensitivity to prosodic cues (Tiryakiol et al., 2026). This pattern illustrates that the early availability of case information can coexist with developmental differences in how linguistic cues are weighted during comprehension. Previous eye-tracking work from our laboratory using the same reversible SOV/OSV materials as the present study, in which the same two animate referents and verb were used across complementary case-marked SOV and OSV versions, provides preliminary evidence for such a distinction. Typically developing children showed a reliable preference for the target picture over the distractor by the second noun phrase in canonical sentences, whereas in non-canonical OSV sentences a reliable target preference emerged later, at the sentence-final verb (Işleyen, 2022). In a separate study using the same eye-tracking paradigm and materials, healthy adults showed a reliable target-picture preference by the second noun phrase in both canonical SOV and non-canonical OSV sentences (Yaman, 2022). Although these findings derive from separate samples and were not directly compared statistically, together they suggest that the availability of case information may not translate into the same time course of non-canonical sentence interpretation in children and adults. This leaves open how the neural responses to canonical and non-canonical argument configurations are expressed across childhood relative to adulthood, motivating the present ERP comparison of the two age groups.

### 1.2 The P600 and sentence processing

Event-related potentials (ERPs) provide a temporally sensitive measure of the processes involved in sentence comprehension. One ERP component frequently associated with syntactic and morphosyntactic processing is the P600, a late positive-going response that is typically maximal over centro-parietal/posterior scalp sites and is commonly observed from approximately 500 ms onward (Hagoort et al., 1993; Kaan et al., 2000; Osterhout and Holcomb, 1992).

Although the P600 was first observed prominently in response to syntactic violations and garden-path constructions, subsequent work has shown that late positivities can also occur when grammatically well-formed sentences place increased demands on syntactic integration or sentence-level representation (Kaan et al., 2000; Brouwer et al., 2012, 2017). Kaan et al. (2000), for example, elicited a P600 in grammatical, non-garden-path sentences at a position where a long-distance dependency had to be integrated, leading them to propose that the component is sensitive to syntactic integration difficulty. P600 effects have likewise been reported at positions where syntactic dependencies are established or completed in otherwise grammatical sentences (Bulut et al., 2018; Gouvea et al., 2010; Phillips et al., 2005).

Accordingly, the functional interpretation of the P600 has broadened beyond a narrowly defined error-repair response. Different accounts have related late positivities to syntactic reanalysis and repair (Hagoort et al., 1993; Osterhout and Holcomb, 1992), difficulty in establishing syntactic relations (Kaan et al., 2000), the construction and revision of syntactic dependencies (Gouvea et al., 2010), or more general processes involved in constructing, revising, and updating sentence-level representations (Brouwer et al., 2012, 2017). For the present study, the important common point is that increased processing demands can elicit late positive activity even when the sentence is grammatical. This makes the P600 relevant to the canonical SOV–non-canonical OSV contrast in Turkish: both sentence types are well-formed and morphologically disambiguated, so any difference in late positivity can be examined in relation to the additional demands associated with processing the non-canonical configuration rather than a grammatical violation.

The sentence-final verb provides a particularly informative point at which to examine these processing demands. In Turkish verb-final sentences, case marking allows thematic-role information to be used incrementally before the verb is encountered (Özge et al., 2019). The verb nevertheless supplies the predicate and its argument-structure information, allowing the previously encountered case-marked arguments to be integrated into a clause-level event representation. Behavioral evidence from Turkish further identifies the sentence-final verb as an informative locus of both processing difficulty and individual variation in the canonical–non-canonical contrast. In a self-paced reading study of reversible SOV and OSV sentences, adult participants read non-canonical OSV sentences more slowly than canonical SOV sentences both at the first noun phrase, where case marking first distinguished the two structures, and at the sentence-final verb (Bulut and Günhan Senol, 2026). Critically, however, individual differences in visual statistical learning modulated the SOV–OSV reading-time difference at the verb but not at the first noun phrase or the intervening regions. Thus, although processing difficulty was detectable as soon as the initial case cue became available, the verb constituted a later locus at which the canonicality effect was also related to individual variation. This is particularly relevant to the present study, which examines group-level ERP effects and whether verb-locked neural responses vary with age and language-related abilities rather than statistical learning. Comparable electrophysiological evidence comes from Japanese, another verb-final language permitting SOV–OSV scrambling. Yano and Koizumi (2018) found a larger P600 for OSV than SOV at the verb in one discourse condition, relating the effect to the operations involved in thematic-role assignment and the integration of the verb with its arguments. Together, these findings provide converging behavioral and electrophysiological motivation for examining late neural activity at the verb as an index of the processing demands associated with non-canonical OSV word order.

### 1.3 Development of morphosyntactic ERP responses

Sensitivity to syntactic and morphosyntactic structure is evident in ERPs from early childhood, but the resulting late positive responses are not necessarily identical to those observed in adults. Two-year-old children have been shown to differentiate phrase-structure violations electrophysiologically, including a late positive response that was delayed and more prolonged relative to the adult response (Oberecker et al., 2005). Similarly, morphosyntactic anomalies elicited late positivities in 3- and 4-year-old children, with substantial age-related differences in the timing and scalp distribution of these responses even across this relatively narrow age range (Silva-Pereyra et al., 2005). P600-like sensitivity to syntactic structure has also been reported at 24 months, although with a later time course than is typically observed in adults (Brusini et al., 2016). Together, these findings indicate that neural sensitivity to syntactic structure emerges early, while the temporal and spatial characteristics of the associated late positivity continue to differ across childhood.

Evidence from school-age children likewise suggests continued age-related differences in the P600, although the pattern across studies is not uniform. In a cross-sectional comparison spanning 6- to 13-year-old children and adults, Hahne et al. (2004) found late positive responses to phrase-structure violations across childhood, but the responses of younger children were generally later and less pronounced, with the 6-year-old group showing a particularly delayed effect. Consistent with continued differences during the school years, Schneider and Maguire (2019) reported age-related differences in the amplitude and scalp distribution of the P600 during naturally paced auditory sentence processing in 8- to 9-year-olds, 12- to 13-year-olds, and adults. Other findings, however, point to substantial adult-like P600 characteristics during childhood: Atchley et al. (2006) observed a P600 to syntactic violations in children approximately 8–13 years of age that was similar to the adult response in latency, amplitude, and scalp distribution. Thus, the developmental literature does not support a simple progression from an absent child P600 to an adult-like response. Rather, age-related differences can involve the timing, magnitude, and topography of the late positivity, and their expression appears to depend on the linguistic manipulation and experimental context (Atchley et al., 2006; Hahne et al., 2004; Schneider and Maguire, 2019). The studies reviewed here predominantly concern typically developing samples, but they span only a limited range of languages and speaker populations. Their developmental patterns should therefore not be treated as universal benchmarks; broader cognitive-science work has cautioned that over-reliance on narrow linguistic samples can lead to overgeneralization (Blasi et al., 2022).

Much of this developmental literature has contrasted well-formed sentences with overt syntactic or morphosyntactic violations (Atchley et al., 2006; Hahne et al., 2004; Oberecker et al., 2005; Silva-Pereyra et al., 2005), but a smaller body of work has examined grammatical non-canonical argument structures and case-based thematic-role assignment. In German, Schipke et al. (2012) compared grammatical case-marked subject-initial and object-initial sentences in 3-year-olds, 4; 6-year-olds, 6-year-olds, and adults. The ERP response to object-initial structures changed markedly across development: 3-year-olds showed a positivity at the initial object, 4; 6-year-olds showed a positivity at the second noun phrase, and 6-year-olds displayed the adult-like initial negativity but continued to show a pronounced later positivity at the second noun phrase, interpreted as reflecting increased effort in syntactic integration and thematic-role assignment; this later positivity was absent in adults. Related work with 2- and 3-year-old German children likewise demonstrates early electrophysiological sensitivity to case-marked subject- and object-initial structures, while showing that the weighting and integration of syntactic case and semantic animacy cues remain developmentally distinct from adults (Strotseva-Feinschmidt et al., 2019). Evidence from Russian further indicates developmental change in late positive responses during grammatical thematic-role assignment: in 4- to 6-year-olds processing reversible active and passive sentences, sensitivity to the relevant inflectional cues was evident across age groups, whereas a P600 was observed only in the 6-year-old group (Kruchinina et al., 2022).

Together, these studies show that developmental differences in neural sentence processing are not confined to responses to grammatical violations: they also arise when children must use morphosyntactic cues to interpret well-formed non-canonical argument configurations. Nevertheless, this evidence remains relatively limited and comes primarily from other case-marking languages. Thus, adult–child ERP comparisons of grammatical, morphologically marked constructions are not unprecedented. However, it remains unknown how grammatical, case-disambiguated SOV and OSV sentences are represented electrophysiologically across development in Turkish, where non-canonical OSV order changes constituent order without rendering the sentence ungrammatical.

### 1.4 Age and grammatical ability in children's sentence processing

Chronological age provides an important index of developmental differences in sentence processing, but it is necessarily a broad and non-specific index: with increasing age, children accumulate linguistic experience while also undergoing cognitive and neurophysiological development. Children of similar ages can differ substantially in their grammatical ability, and neuroimaging evidence indicates that such differences can account for variation in syntactic processing beyond that associated with age. In German preschool children, for example, neural responses to case-marked canonical subject-initial and non-canonical object-initial sentences varied with receptive grammatical knowledge; the use of case-marking cues for sentence interpretation was more closely related to children's grammatical knowledge than to chronological age alone (Knoll et al., 2012). Similarly, in school-age children and adolescents, better syntactic skills were associated with greater recruitment of left inferior frontal regions during syntactic processing even after chronological age was taken into account (Nuñez et al., 2011). Thus, chronological age and grammatical ability appear to capture related but partly distinguishable sources of variation in children's sentence processing.

Relevant evidence also comes from Turkish. Özkan et al. (2022) examined 4- to 8-year-old children's anticipatory use of nominative and accusative case marking during verb-final sentence comprehension while controlling for chronological age. Children's morphosyntactic prediction was related to early productive vocabulary, current language-production ability, and episodic-buffer performance, whereas receptive grammar measured at the time of testing was not a significant predictor. These findings indicate that chronological age alone does not capture all individual variation in Turkish children's use of case information, while also showing that not every concurrent language measure necessarily contributes independently. In particular, it is not known whether grammatical ability predicts children's comprehension or neural processing of grammatical canonical and non-canonical word orders beyond age.

Comparable individual differences have been observed in electrophysiological measures of syntactic processing. (Wray and Weber-Fox 2013) found that, among typically developing 7- and 8-year-old children, grammatical morphology proficiency was associated with the timing and scalp distribution of the P600 elicited by phrase-structure violations, including an earlier onset and a more sustained posterior response in children with stronger grammatical proficiency. Related variation is also evident in adulthood: in monolingual native-speaking adults, Pakulak and Neville (2010) found systematic differences in ERP responses to phrase-structure violations as a function of standardized language proficiency, including a larger and more broadly distributed P600 in higher-proficiency speakers. These findings motivate examining whether grammatical ability accounts for variation in late morphosyntactic ERP responses beyond chronological age.

However, the available ERP evidence relating grammatical ability to late positive responses has predominantly come from paradigms involving syntactic or morphosyntactic violations (Pakulak and Neville, 2010; Wray and Weber-Fox, 2013). Much less is known about whether children's grammatical ability accounts for variation in electrophysiological processing when both alternatives are well formed but differ in canonicity and processing demands. This question is particularly relevant for Turkish, where comprehension of non-canonical OSV sentences requires the effective use of morphosyntactic case cues despite departure from the dominant SOV order. Examining chronological age and grammatical ability jointly therefore allows us to ask whether individual variation in children's behavioral and neural responses to canonical and non-canonical word order is associated with broad age-related developmental differences, with their current grammatical ability, or with both.

### 1.5 The present study

Taken together, the literature reviewed above provides several converging lines of evidence relevant to the present study. Turkish-speaking adults show measurable processing costs for non-canonical OSV relative to canonical SOV word order (Ergin and Stromswold, 2023; Bulut and Günhan Senol, 2026), while Turkish-speaking children can use case marking incrementally to assign thematic roles during sentence comprehension (Özge et al., 2019). Cross-linguistic ERP studies further show that grammatical non-canonical argument structures can elicit late positive responses and that the neural processing of such structures continues to differ across childhood (Schipke et al., 2012; Yano and Koizumi, 2018). However, it remains unclear how grammatical, case-disambiguated SOV and OSV sentences are processed electrophysiologically in Turkish-speaking children and adults. To our knowledge, no published study has directly compared Turkish-speaking children and adults using this particular grammatical SOV–OSV contrast. It is also unknown whether variability in children's processing of these canonical and non-canonical structures is associated with chronological age, grammatical ability, or both.

The present study addressed these questions by comparing the comprehension of canonical SOV and grammatical non-canonical OSV sentences [as illustrated above in (1)] in Turkish-speaking children and adults. The two conditions contained the same nouns and verb, with both possible agent–patient assignments being semantically plausible; thematic roles therefore had to be recovered from morphosyntactic cues rather than lexical-semantic plausibility. ERPs were time-locked to the sentence-final verb, a theoretically important point at which the preceding case-marked arguments can be integrated with the predicate. As reviewed above, this focus was supported by converging behavioral, eye-tracking, and ERP evidence identifying the verb as an informative locus of processing difficulty and individual variation in non-canonical sentence processing (Işleyen, 2022; Yaman, 2022; Yano and Koizumi, 2018; Bulut and Günhan Senol, 2026). By combining behavioral accuracy with verb-locked ERPs, we therefore examined whether the additional processing demands associated with non-canonical OSV word order were expressed at both the behavioral and neural levels. Based on the strong predominance of SOV over OSV in Turkish (Türk et al., 2022), previous behavioral evidence for a canonical processing advantage in Turkish (Ergin and Stromswold, 2023; Bulut and Günhan Senol, 2026), and evidence that non-canonical OSV word order can elicit late positive activity at the verb in another verb-final language (Yano and Koizumi, 2018), we expected non-canonical OSV sentences to be more demanding than canonical SOV sentences. Behaviorally, we expected lower comprehension accuracy for non-canonical OSV than canonical SOV sentences. Electrophysiologically, we expected non-canonical OSV sentences to elicit a greater late posterior positivity than canonical SOV sentences. We also examined whether the magnitude of the SOV–OSV difference varied between children and adults at both the behavioral and ERP levels.

Three related aims were addressed. First, we compared canonical SOV and non-canonical OSV sentence processing in children and adults at the behavioral and ERP levels, asking whether the magnitude of the SOV–OSV difference varied between the two groups. Second, within children, we examined whether chronological age was associated with behavioral performance or with the neural response to sentence type. Third, because chronological age and grammatical ability capture related but partly distinguishable sources of variation in children's sentence processing (Knoll et al., 2012; Wray and Weber-Fox, 2013), we tested whether grammatical ability accounted for variation in behavioral and ERP responses over and above age. Together, these analyses allowed us to examine group-level differences between children and adults alongside continuous age- and grammar-related variation within childhood in a contrast where both sentence structures were grammatical and interpretation depended on morphosyntactic case cues.

## 2 Methods

The study was approved by the Istanbul Medipol University Non-Interventional Clinical Research Ethics Committee (Decision No. 721, 25 September 2019). Written informed consent was obtained from all adult participants and from the parents of participating children.

### 2.1 Participants

The sample comprised 104 native speakers of Turkish: 67 adults and 37 children. The adult group included 45 women and 22 men and ranged in age from 18 to 44 years (*M* = 29.21, *SD* = 9.21). Adult educational attainment was primary school for three participants (4.5%), secondary school for five (7.5%), high school for 34 (50.7%), and university or higher for 25 (37.3%). Years of formal education for adults were as follows: *M* = 14.10, *SD* = 4.09, range = 5–22 years. The child group included 13 girls and 24 boys and ranged in age from 4.33 to 9.67 years (*M* = 6.83, *SD* = 1.20; age in months: *M* = 81.97, *SD* = 14.41, range = 52–116). Thus, the child sample covered a relatively broad developmental span from preschool to primary-school age. Adults self-reported, and parents reported for children, no diagnosed neurological, cognitive, motor, or psychiatric disorders and no known uncorrected hearing or visual impairments. Behavioral response data were unavailable for two children; behavioral analyses therefore included 35 children, whereas ERP analyses included all 37.

### 2.2 Stimuli

#### 2.2.1 Sentence materials

This study employed an auditory sentence–picture matching task comprising canonical SOV, non-canonical OSV, and filler sentences. Forty-eight sentence sets were constructed, each with a canonical SOV and a non-canonical OSV version containing the same two nouns and sentence-final transitive verb (Table 1). The complete set of canonical SOV and non-canonical OSV experimental sentences, together with their English translations, is provided in Supplementary Table S1. The 96 sentences were distributed across two complementary lists: each list contained one version of every sentence set, with 24 canonical SOV and 24 non-canonical OSV sentences, whereas the alternative version of each set appeared in the other list. Both lists were administered as separate blocks. Participants completing both blocks therefore heard 48 canonical SOV and 48 non-canonical OSV sentences in total.

**Table 1:** Example sentence materials used in the ERP experiment.

Sentence type	NP1	NP2	Verb
Canonical SOV (*n* = 48)	Polis _AGENT_	Doktoru _PATIENT_	Boyuyor
	Police officer	Doctor-ACC	Paint-PROG.3SG
	The police officer is painting the doctor		
Non-canonical OSV (*n* = 48)	Polisi _PATIENT_	Doktor _AGENT_	Boyuyor
	Police officer-ACC	Doctor	Paint-PROG.3SG
	The doctor is painting the police officer		

ACC, accusative case; NP, noun phrase; PROG, progressive; 3SG, third person singular.

Each canonical SOV and non-canonical OSV sentence contained two animate, profession-denoting nouns, both of which were plausible agents of the action. The use of two animate referents therefore made the thematic relations reversible and prevented animacy from identifying the agent and patient. Canonical SOV sentences followed the subject–object–verb order, whereas non-canonical OSV sentences followed an object–subject–verb order. Within each set, the lexical NP sequence and the verb were held constant across the two versions. In canonical SOV sentences, NP1 was the nominative subject and NP2 the accusative object, yielding SOV order; in non-canonical OSV sentences, accusative marking occurred on NP1 and NP2 was the nominative subject, yielding OSV order. The two versions therefore expressed the two complementary agent–patient assignments using the same lexical material. Because either referent could plausibly perform the action, semantic information alone was insufficient to identify the agent and patient. Successful thematic-role assignment therefore depended on resolving morphosyntactic cues rather than lexical-semantic plausibility.

Developmental appropriateness and visual identifiability were explicit criteria during stimulus preparation. To evaluate these criteria, the lexical items and corresponding pictures were reviewed by a research team comprising one PhD candidate and one master's student in Speech and Language Therapy and one researcher with a PhD in cognitive neuroscience, with additional input from three speech-language therapists with PhDs and professional experience in speech and language intervention with children. Candidate lexical items were evaluated for expected familiarity to Turkish-speaking children from approximately 4 years of age onward and for ease of visual representation and identification.

The 48 sentence sets were constructed from a fixed lexical pool of 12 profession-denoting nouns and 12 transitive verbs. The canonical SOV and non-canonical OSV versions of each set contained the same noun and verb lemmas and were therefore matched exactly in lexical frequency and lexical length by design. Given the rich inflectional morphology of Turkish, lexical frequency was characterized using lemma frequency, which counts occurrences of a lexical item across its inflected forms rather than occurrences of a single surface word form (Eskioglu et al., 2025). The nouns had a mean length of 6.50 phonemes (range = 4–9) and a mean lemma frequency of 98.12 occurrences per million words (range = 1.85–499.70), whereas the verbs had a mean length of 4.50 phonemes (range = 2–7) and a mean lemma frequency of 238.93 occurrences per million words (range = 4.34–1046.35). These frequencies were obtained from the entire Turkish National Corpus (TNC; Aksan et al., 2012), which includes both written and spoken language and comprises 49,664,176 written and 1,014,023 spoken words. Given the auditory presentation of the stimuli and the comparatively limited and developmentally variable print exposure of the child sample due to their age, lemma frequencies were additionally calculated using the spoken subcorpus alone. In the spoken subcorpus, the nouns had a mean lemma frequency of 88.92 occurrences per million words (range = 0.99–411.23). Eleven of the 12 verbs were attested in the spoken subcorpus, with a mean lemma frequency of 198.76 occurrences per million words (range = 5.92–947.71); one of the verbs used in the present study, *tekmele* (“kick”), was not attested in the spoken subcorpus. Because the TNC is a general-purpose corpus rather than a child-directed or age-stratified corpus, these frequencies were treated as descriptive indices of lexical occurrence rather than as direct measures of lexical familiarity in young children. Item-level full-corpus and spoken-subcorpus frequencies, together with details of the corpus-search procedure, are provided in Supplementary Table S20 and the accompanying Supplementary material text.

To provide an independent retrospective developmental estimate of lexical accessibility, subjective age-of-acquisition (AoA) ratings were subsequently collected for the 24 experimental lexical items from 137 Turkish-speaking adults (125 women, age *M* = 19.51 years, *SD* = 1.74, range = 18–35 years) using a procedure adapted from Kuperman et al. (2012). Ratings for 24 control words showed a very strong rank-order association with previously published independent Turkish subjective AoA norms (Eskioglu et al., 2025), Spearman's ρ = 0.981, *p* < 0.001. Across the experimental items, mean rated AoA was 3.51 years for the verbs and 5.18 years for the nouns; median item AoAs ranged from 2 to 7 years. Thus, the retrospective norms indicated relatively early acquisition for many items but did not establish that every lexical item would have been familiar to every child at the youngest ages tested. Full norming procedures and item-level AoA estimates are provided in Supplementary Table S20 and the accompanying Supplementary material text.

The occurrence and position of these lexical items were systematically balanced within each stimulus list. Each noun occurred eight times per list, four times in NP1 and four times in NP2, and each verb occurred four times per list. Each noun pair occurred only once within a list, and a given noun was paired with a given verb only once within a list. Because the two lists contained complementary canonical SOV and non-canonical OSV versions of the same 48 sentence sets, the same noun–verb combinations occurred again in the other list in the alternative sentence-type condition.

The plausibility of the sentences was evaluated in a separate norming study involving 73 adults who did not participate in the main experiment (64 women; age *M* = 19.6 years, *SD* = 1.0). Participants rated, on a 7-point scale, how likely the event described by each sentence was to occur in the real world. Canonical SOV (*M* = 2.91, *SD* = 0.80) and non-canonical OSV sentences (*M* = 2.79, *SD* = 0.73) did not differ significantly in plausibility, *t*_(47)_ = 1.14, *p* = 0.261.

Each stimulus list also contained 24 filler trials (12 SOV and 12 OSV), with complementary versions of the 24 filler sets distributed across the two lists, yielding 48 filler trials across the two-block experiment. Unlike the reversible experimental sentences, the fillers had non-reversible thematic relations, involving an animate agent and an inanimate patient (e.g., *Kaptan pastayi kesiyor*, “The captain is cutting the cake”). Thus, unlike the experimental sentences, their thematic roles could be determined largely from semantic information. The fillers increased the semantic and structural variety of the task and discouraged reliance on a strategy specific to sentences containing two animate referents. They were not included in the analyses reported here.

#### 2.2.2 Audio recordings

All sentences were recorded in a sound-attenuated room by a native speaker of Turkish using a Tascam DR-05 digital recorder. The speaker was instructed to produce the sentences with neutral and consistent prosody. Each sentence was recorded at least twice, and the recording showing the smallest prosodic differences between the corresponding canonical SOV and non-canonical OSV versions was selected for use in the experiment.

The selected recordings were amplitude-normalized in Audacity and segmented into the first noun phrase (NP1), second noun phrase (NP2), and sentence-final verb. Segment durations were measured in Praat (Boersma and Weenink, 2018). To eliminate duration differences between the canonical SOV and non-canonical OSV versions of each sentence set, corresponding segments were adjusted using Praat's overlap-add duration-manipulation procedure. Following adjustment, the two versions of each sentence set had identical NP1, NP2, and verb durations. Across the 48 sentence sets, mean adjusted durations were 751.99 ms (*SD* = 94.25) for NP1, 818.35 ms (*SD* = 81.52) for NP2, and 702.92 ms (*SD* = 83.67) for the verb.

Prosodic differences associated with the sentence-type manipulation were additionally assessed at NP1, the earliest position at which the canonical SOV and non-canonical OSV sentences differed morphosyntactically. Following Hanne et al. (2015), the F0 contour over NP1 was calculated as the difference between maximum and minimum F0. Canonical SOV and non-canonical OSV sentences did not differ significantly on this measure, *t*_(47)_ = 1.55, *p* = 0.128.

#### 2.2.3 Visual materials

Black-and-white line drawings were created by a professional illustrator to accompany the auditory sentences. Black-and-white line drawings were used to keep the visual displays relatively schematic and standardized and to reduce uncontrolled variation in surface features such as color across items, consistent with their common use in psycholinguistic sentence–picture matching paradigms (e.g., Hanne et al., 2015). For each of the 48 canonical SOV–non-canonical OSV sentence sets, two complementary drawings depicted the two possible thematic-role assignments, yielding 96 experimental pictures in total (Figure 1). For example, one drawing depicted the police officer painting the doctor, whereas the other depicted the doctor painting the police officer. Thus, the picture corresponding to the canonical SOV version of a sentence set served as the alternative thematic-role interpretation for the non-canonical OSV version, and vice versa. The same pair of complementary pictures for a given sentence set was presented once in each block, with the target and alternative pictures reversing across the two complementary sentence versions. An additional 24 pictures were prepared for the filler materials.

**Figure 1:**
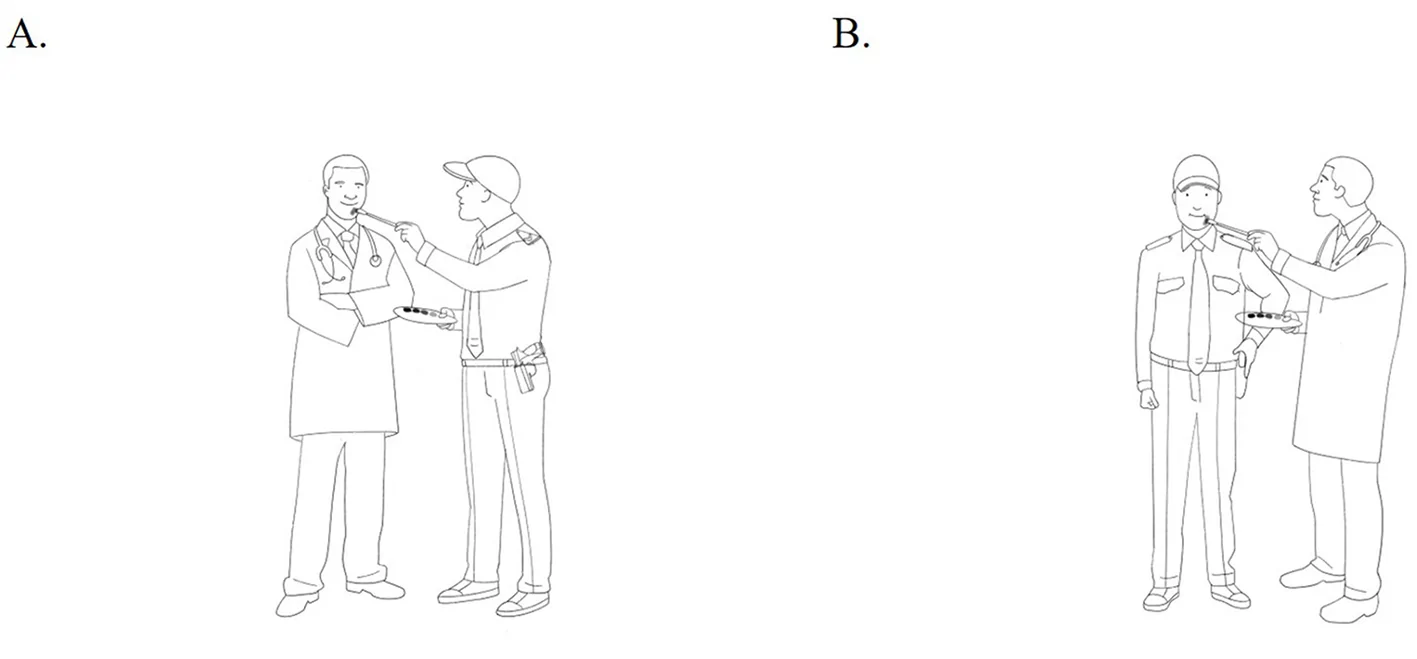
Example target and distractor pictures used in the sentence–picture matching task. The example corresponds to the canonical SOV sentence *Polis doktoru boyuyor* (“The police officer is painting the doctor”). **(A)** Target. **(B)** Distractor.

The illustrations were standardized to minimize visual differences unrelated to sentence interpretation. Recurring characters and actions were depicted as consistently as possible across items; for example, the same profession was represented by a similar-looking character across drawings, and recurring actions were illustrated in a comparable manner. All human figures were male. The drawings were centered within the image frame, occupied a comparable proportion of the available space, and were digitized at the same resolution.

The spatial arrangement of the depicted agent and patient was also balanced across the materials: the patient appeared to the left of the agent in half of the pictures and to the right in the other half. During the task, the two alternative pictures corresponding to a sentence were presented simultaneously as response options, with the position of the target picture balanced across items.

### 2.3 ERP task and procedure

The experimental task was developed in SR Research Experiment Builder and compiled as a standalone executable. Auditory sentences were presented through headphones. Participants were instructed to listen carefully to each sentence and, after sentence presentation, indicate which of two pictures best matched its meaning by pointing to the corresponding picture. Before the main experiment, participants completed four practice trials that were not included in the analyses; additional instructions were provided during practice when necessary.

The experiment comprised two blocks corresponding to the two stimulus lists described above. Each block contained 24 canonical SOV, 24 non-canonical OSV, and 24 filler trials, for a total of 72 trials per block and 144 trials across the experiment. The order of the two blocks was counterbalanced across participants, and trial order was randomized independently within each block. A rest break was provided between the two blocks, with additional breaks given when needed. All adults completed both experimental blocks. Among the children, 30 of 37 completed both blocks, whereas 7 completed one block only.

The ERP procedure with the sequence of events within each trial is illustrated in Figure 2. Each trial initially displayed a blank screen and was started by the experimenter once the participant indicated readiness. A fixation cross then appeared at the center of the screen, and auditory sentence presentation began 500 ms later. The sentence was presented sequentially as NP1, NP2, and the sentence-final verb, with mean durations of approximately 752, 818, and 703 ms, respectively. The fixation cross remained on the screen throughout sentence presentation. No response pictures were visible during auditory sentence presentation.

**Figure 2:**
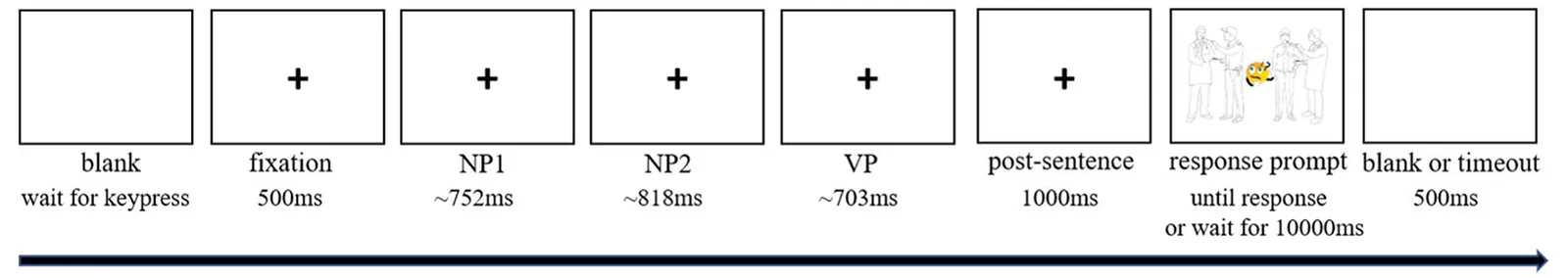
ERP procedure.

Following the offset of the sentence-final verb, the fixation cross remained onscreen for a further 1,000 ms (post-sentence silent period), during which no auditory stimulus was presented. This interval was included to allow measurement of late ERP activity elicited by the verb before presentation of the response pictures. After this interval, the fixation cross disappeared and the two alternative pictures appeared on the left and right sides of the screen, with a question symbol presented between them. Participants had up to 10 s to point to the picture matching the sentence they had heard. The experimenter recorded the response by pressing the corresponding left or right key on the keyboard. The response display was followed by a blank screen for 500 ms before the next trial. The ERP task lasted approximately 45–60 min, depending on the duration of breaks and participant cooperation.

### 2.4 EEG recording and preprocessing

Continuous EEG was recorded using a Brain Products actiCHamp system and actiCAP electrode caps with BrainVision Recorder (Brain Products GmbH, Gilching, Germany) at a sampling rate of 1,000 Hz. The recording montage comprised 27 scalp EEG electrodes, with Fz serving as the online reference, two horizontal electrooculogram (HEOG) electrodes, and two vertical electrooculogram (VEOG) electrodes. A separate electrode served as ground. Electrode impedances were kept below 10 kΩ during recording. Appropriate cap sizes were used for children and adults.

Offline preprocessing was performed in BrainVision Analyzer. The continuous EEG data were first downsampled to 500 Hz and subsequently band-pass filtered from 0.05 to 70 Hz. The data were then rereferenced to the average of the 27 scalp EEG electrodes and segmented into epochs time-locked to the onset of the sentence-final verb, extending from −200 to 1,500 ms relative to verb onset. Ocular artifacts were corrected using independent component analysis (ICA), with the horizontal and vertical EOG channels used for ocular-artifact identification. The epochs were then baseline-corrected using the −200 to 0 ms interval. Epochs containing amplitudes exceeding ±100 μV were rejected, and the remaining epochs were averaged separately for the canonical SOV and non-canonical OSV conditions, irrespective of subsequent behavioral response accuracy.

Because artifact rejection was channel-specific, the number of retained epochs varied slightly across electrodes. Across the six electrodes included in the final ERP analyses (P3, Pz, P4, O1, Oz, and O2), adults retained an average of 99.4% of available canonical SOV epochs and 99.7% of available non-canonical OSV epochs. For children, retention was calculated relative to the number of trials actually presented to each participant, thereby accounting for those who completed only one experimental block. Children retained an average of 91.4% of available canonical SOV epochs and 88.6% of available non-canonical OSV epochs. Electrode-specific epoch-retention statistics are provided in Supplementary Table S2.

### 2.5 Child language and additional individual-difference measures

Children's language abilities were assessed using the Türkçe Okul Çagi Dil Gelişim Testi (Turkish School-Age Language Development Test; TODIL; Topbaş and Güven, 2017), a standardized, norm-referenced language assessment for Turkish-speaking children aged 4; 0–8; 11 years. TODIL includes six core and three supplementary subtests. The six core subtests were administered in the present study: picture vocabulary, relational vocabulary, oral vocabulary, syntactic understanding, sentence imitation, and morphological completion.

For the present analyses, raw subtest scores were standardized within the participating child sample rather than converted to age-normed TODIL scores. A grammar composite, used as an index of grammatical ability, was calculated as the mean of the standardized scores for syntactic understanding, sentence imitation, and morphological completion, and an overall language composite as the mean of the standardized scores across all six core subtests. Accordingly, the published age norms were not used in the analyses, including for children older than the upper limit of the normative age range.

Forward digit span (FDS) was additionally administered as a measure of verbal short-term memory and was available for 21 of the 37 children. The examiner presented sequences of digits auditorily at a rate of approximately one digit per second, and children were asked to repeat the digits in the same order. The task began with sequences of three digits and increased by one digit at each successive level. Two trials were administered at each sequence length. Testing proceeded to the next level if the child correctly recalled at least one of the two trials and was discontinued when both trials at a given sequence length were recalled incorrectly. Forward-digit-span level was defined as the longest sequence length at which at least one of the two trials was recalled correctly.

Maternal and paternal educational attainment was available for all 37 children and was coded into four ordered categories: primary, secondary, high school, and university and above. Descriptive statistics for the child measures and parental educational attainment are provided in Supplementary Table S3.

### 2.6 Data analysis

Statistical analyses were conducted in R version 4.5.1 (R Core Team, 2025).

#### 2.6.1 Behavioral analyses

**Main analyses**. Behavioral performance was quantified for each participant as percentage accuracy separately for canonical SOV and non-canonical OSV sentences. First, these participant-level accuracy percentages were analyzed using linear mixed-effects regression models with a random intercept for participant to account for repeated observations across sentence types. The primary model included Group (adult, child), Sentence Type (canonical SOV, non-canonical OSV), and their interaction as fixed effects. Planned follow-up contrasts compared canonical SOV and non-canonical OSV accuracy separately within adults and children, with Holm correction across the two within-group comparisons. Adult–child differences were also examined separately for the canonical SOV and non-canonical OSV conditions, with Holm correction across these two comparisons. The between-group difference in the SOV – OSV accuracy difference constituted a single planned contrast and was therefore not additionally corrected for multiple comparisons. Second, developmental and language-related analyses were conducted within the child group. Age in years was mean-centered prior to analysis. Age-related differences in behavioral performance were first examined in a model including Sentence Type, Age, and their interaction. To determine whether the grammar composite accounted for variation in performance beyond age, a subsequent model included Sentence Type × Age and Sentence Type × Grammar composite terms. An analogous model replacing the grammar composite with the overall language composite was conducted as a robustness analysis. For these models, condition-specific age or language slopes and the difference between the canonical SOV and non-canonical OSV slopes were estimated to determine whether associations with behavioral performance differed across sentence types.

**Sensitivity and exploratory analyses**. First, to examine whether the primary group-level behavioral findings were robust to restricting the sample to children with less uncertainty regarding lexical accessibility, the Group × Sentence Type analysis was repeated after restricting the child group to participants aged 84 months (7 years) or older. This cutoff was selected because 7 years was at or above the median retrospective AoA of every experimental lexical item (Supplementary Table S20). The restriction retained 18 children and all 67 adults, and the same model, planned contrasts, and Holm correction procedures as in the primary Group × Sentence Type analysis were used. Second, to examine whether age-related associations might be non-linear, a sensitivity model added a quadratic age term and its interaction with Sentence Type to the linear age model. Behavioral accuracy and the SOV – OSV accuracy difference were also summarized descriptively for children aged 4–5, 6–7, and 8–9 years. Third, verbal short-term memory was examined in a sensitivity analysis using forward digit span. Analyses involving this measure were restricted to children with available forward-digit-span data; 20 of the 21 children with such data also had behavioral responses. Forward-digit-span scores were standardized within the available child sample. The behavioral model examined the association between forward digit span and comprehension accuracy while controlling for age, as well as whether this association differed between canonical SOV and non-canonical OSV sentences. The grammar model was then re-estimated in the same 20 children with and without forward digit span as an additional covariate to assess whether the grammar–comprehension association was robust to adjustment for verbal short-term memory. Fourth, to examine the potential contribution of family educational background, maternal educational attainment was examined in additional sensitivity analyses. Maternal education was coded from 1 (primary) to 4 (university and above) and mean-centered for inferential analyses. Its association with the grammar composite was first assessed while controlling for age. The behavioral grammar model was then repeated with maternal education included as an additional covariate to determine whether the grammar–comprehension association remained after adjustment for maternal educational background. Paternal education was retained for descriptive purposes only. Finally, an exploratory analysis examined the TODIL morphological-completion subtest as an alternative individual-difference predictor. The morphological-completion score was standardized within the child sample and substituted for the grammar composite in an otherwise analogous behavioral model. Because morphological completion is itself a component of the grammar composite, the two predictors were not entered simultaneously. All behavioral inferential tests, including the main analyses as well as the sensitivity and exploratory analyses, were two-tailed.

#### 2.6.2 ERP analyses

**Main analyses**. ERP analyses were based on mean amplitudes calculated from all artifact-free epochs, irrespective of behavioral response accuracy. Analyses focused on the prespecified 600–1,200-ms interval following sentence-final verb onset. This interval and the posterior region of interest (ROI) were selected on the basis of previous work showing late syntactic positivities with predominantly centro-parietal or posterior distributions (Hagoort and Brown, 2000; Hahne et al., 2004; Kaan et al., 2000), including a posterior positivity associated with non-canonical OSV relative to canonical SOV word order in another verb-final language (Yano and Koizumi, 2018). The interval was extended to 1,200 ms to accommodate the later timing of syntactic ERP responses reported in children (Hahne et al., 2004). To characterize the time course of the response without assuming identical timing across adults and children, mean amplitudes were calculated in consecutive 100-ms windows, an approach previously used to examine developmental and individual variation in late syntactic positivities (Wray and Weber-Fox, 2013). Mean amplitudes were therefore calculated separately for six consecutive 100-ms time windows (600–700, 700–800, 800–900, 900–1,000, 1,000–1,100, and 1,100–1,200 ms) within a posterior ROI comprising P3, Pz, P4, O1, Oz, and O2.

First, group differences in ERP responses were examined using separate linear mixed-effects models for each of the six time windows. Each model included Group (adult, child), Sentence Type (canonical SOV, non-canonical OSV), and their interaction as fixed effects and a random intercept for participant. Residual variances were allowed to differ across the four Group × Sentence Type cells. Omnibus tests of Group, Sentence Type, and Group × Sentence Type were two-tailed, with Benjamini–Hochberg false discovery rate (BH-FDR) correction performed separately for each model term across the six time-window models. To test the hypothesized greater late positivity for non-canonical OSV than canonical SOV sentences, OSV – SOV contrasts were estimated separately within adults and children. These within-group contrasts were evaluated using one-tailed directional tests of OSV > SOV, with BH-FDR correction conducted separately within each group across the six time windows. Adult–child differences in the OSV – SOV effect, corresponding to the Group × Sentence Type interaction, were evaluated using two-tailed tests with BH-FDR correction across the six interactions. As secondary analyses, adult–child differences in ERP amplitude were also examined separately within the canonical SOV and non-canonical OSV conditions; these tests were two-tailed, with BH-FDR correction across all 12 condition-specific comparisons.

Second, developmental ERP analyses were conducted within the child group using the same posterior ROI and six time windows. Age in years was mean-centered prior to analysis. Separate models were fitted for each time window with Sentence Type, Age, and their interaction as fixed effects and a random intercept for participant; residual variance was allowed to differ between sentence types. All age-related tests were two-tailed. For the omnibus analyses, BH-FDR correction was performed separately for each model term across the six time-window models. Condition-specific age slopes were corrected together across the 12 Sentence Type × time-window estimates, whereas the six Sentence Type × Age interactions were corrected separately. To examine whether the grammar composite accounted for variation in ERP amplitude beyond age, analogous child-only models included Sentence Type × Age and Sentence Type × Grammar composite terms. Age was therefore retained as a covariate together with its interaction with Sentence Type. All tests were two-tailed, and BH-FDR correction was conducted separately across the six grammar main effects, the six Sentence Type × Grammar interactions, and the 12 condition-specific grammar slopes. The same analyses were repeated using the overall-language composite as a robustness analysis, with corresponding BH-FDR correction applied separately across the six overall-language main effects, six Sentence Type × Overall Language interactions, and 12 condition-specific overall-language slopes.

**Sensitivity and exploratory analyses**. First, as a lexical-accessibility sensitivity analysis, the Group × Sentence Type ERP analyses were repeated after restricting the child group to participants aged 84 months (7 years) or older (*n* = 18), while retaining all 67 adults. As in the primary ERP analyses, models were fitted separately for the six prespecified posterior time windows. Within-child OSV – SOV contrasts were evaluated using one-tailed directional tests of OSV > SOV, with BH-FDR correction across the six time windows, and adult–child differences in the OSV – SOV effect were evaluated using two-tailed tests with BH-FDR correction across the six interactions. Second, to examine whether variation in children's comprehension accuracy was related to their ERP amplitudes, a participant-level sensitivity analysis was conducted in the 35 children with available behavioral data. Within each sentence type, condition-specific accuracy was paired with ERP amplitude from the corresponding condition. Accuracy was centered and expressed per 10-percentage-point increase, and the models included Sentence Type, Age, accuracy, and the interactions of Sentence Type with Age and accuracy. This analysis therefore assessed whether condition-specific comprehension accuracy was associated with ERP amplitude and whether that association differed between canonical SOV and non-canonical OSV sentences. BH-FDR correction was applied separately across the six accuracy main effects and the six Sentence Type × Accuracy interactions, while the 12 condition-specific accuracy slopes were corrected together.

Third, to assess whether the child ERP findings were affected by differences in data availability or task completion, the within-child OSV – SOV contrasts were repeated in two restricted samples: the 35 children with available behavioral data and the 30 children who completed both experimental blocks. The same one-tailed directional OSV > SOV tests were used, with BH-FDR correction across the six time windows separately within each restricted sample.

Fourth, to examine whether developmental associations might be non-linear, the child age models were extended by adding a quadratic age term and its interaction with Sentence Type. BH-FDR correction was applied separately across the six Age^2^ effects and the six Sentence Type × Age^2^ interactions. Posterior OSV – SOV amplitudes were also summarized descriptively for children aged 4–5, 6–7, and 8–9 years.

Finally, an exploratory analysis examined the TODIL morphological-completion subtest as an alternative individual-difference predictor of children's ERP amplitudes. The morphological-completion score was standardized within the child sample and substituted for the grammar composite in otherwise analogous child-only ERP models that retained age and the Sentence Type × Age interaction. Because morphological completion is itself a component of the grammar composite, the two predictors were not entered simultaneously. BH-FDR correction was applied separately across the six morphological-completion main effects, the six Sentence Type × Morphological Completion interactions, and the 12 condition-specific morphological-completion slopes. All tests in this exploratory analysis were two-tailed.

## 3 Results

### 3.1 Group differences in comprehension accuracy for canonical and non-canonical word order

Descriptive statistics for sentence-comprehension accuracy are presented in Supplementary Table S4 and Figure 3. Accuracy was lower for non-canonical OSV than canonical SOV sentences in both groups, with a larger descriptive difference among children. Adults achieved mean accuracies of 96.64% (*SD* = 3.42) for canonical SOV and 93.87% (*SD* = 7.50) for non-canonical OSV sentences, whereas the corresponding means for children were 76.56% (*SD* = 16.78) and 67.65% (*SD* = 15.71), respectively.

**Figure 3:**
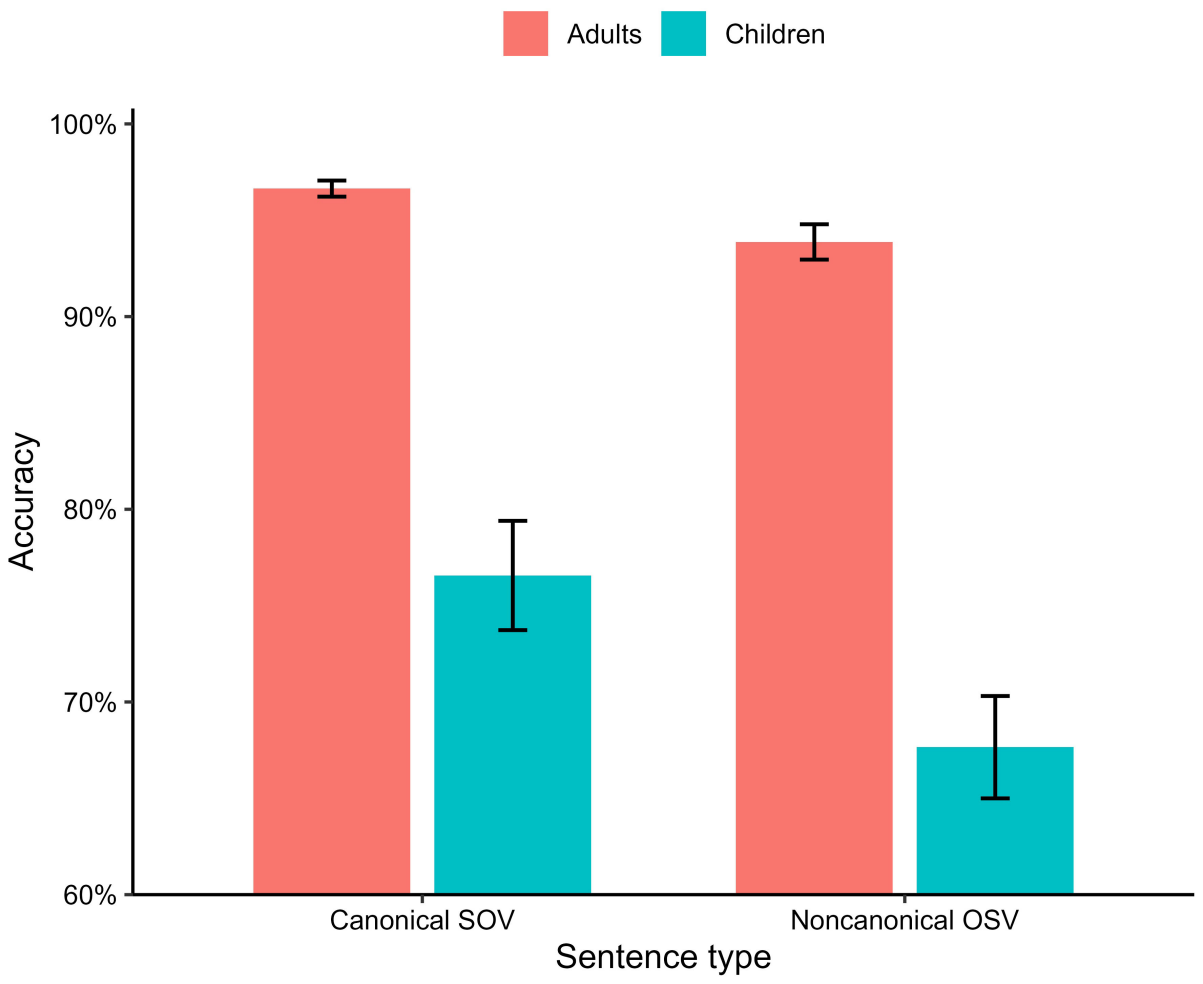
Mean comprehension accuracy for canonical SOV and non-canonical OSV sentences in adults and children. Error bars represent ±1 standard error of the mean.

The Group × Sentence Type mixed-effects model revealed significant main effects of group, *F*_(1, 100)_ = 131.26, *p* < 0.001, and sentence type, *F*_(1, 100)_ = 42.63, *p* < 0.001. These effects were qualified by a significant Group × Sentence Type interaction, *F*_(1, 100)_ = 11.80, *p* < 0.001. Full model results and planned contrasts are provided in Supplementary Table S5.

Planned two-tailed contrasts showed that accuracy was significantly lower for non-canonical OSV than canonical SOV sentences in adults, *b* = 2.77 percentage points, *SE* = 1.05, 95% CI [0.69, 4.85], *t*_(100)_ = 2.64, *p* = 0.010, and children, *b* = 8.91 percentage points, *SE* = 1.45, 95% CI [6.04, 11.79], *t*_(100)_ = 6.15, *p* < 0.001. Adults were also more accurate than children for both canonical SOV sentences, *b* = 20.08 percentage points, *SE* = 2.21, 95% CI [15.71, 24.45], *t*_(137.74)_ = 9.09, *p* < 0.001, and non-canonical OSV sentences, *b* = 26.22 percentage points, *SE* = 2.21, 95% CI [21.85, 30.59], *t*_(137.74)_ = 11.87, *p* < 0.001. The SOV – OSV accuracy difference was 6.14 percentage points larger in children than in adults, *SE* = 1.79, 95% CI [2.60, 9.69], *t*_(100)_ = 3.43, *p* < 0.001. Thus, non-canonical OSV word order was associated with lower comprehension accuracy in both groups, and this accuracy disadvantage was significantly larger in children.

The age-restricted sensitivity analysis yielded the same behavioral pattern (Supplementary Table S21). Among children aged 7 years or older (*n* = 18; age *M* = 7.83 years, *SD* = 0.77, range = 7.00–9.67), mean accuracy was 76.06% for canonical SOV and 67.82% for non-canonical OSV sentences. The SOV – OSV accuracy difference remained significant, *b* = 8.24 percentage points, *SE* = 1.90, 95% CI [4.47, 12.01], *t*_(83)_ = 4.34, Holm-adjusted *p* < 0.001. The Group × Sentence Type interaction also remained significant, *F*_(1, 83)_ = 6.56, *p* = 0.012, and the SOV – OSV difference was 5.47 percentage points larger in the age-restricted child group than in adults, *SE* = 2.14, 95% CI [1.22, 9.72], *t*_(83)_ = 2.56, *p* = 0.012.

### 3.2 Group differences in ERP responses to canonical and non-canonical word order

Grand-average ERP waveforms for canonical SOV and non-canonical OSV sentences at the posterior ROI are shown in Figure 4. Scalp distributions of the non-canonical OSV – canonical SOV difference across the six 100-ms analysis windows are shown in Figure 5. Mean non-canonical OSV – canonical SOV amplitude differences at the posterior ROI across these windows are shown in Figure 6.

**Figure 4:**
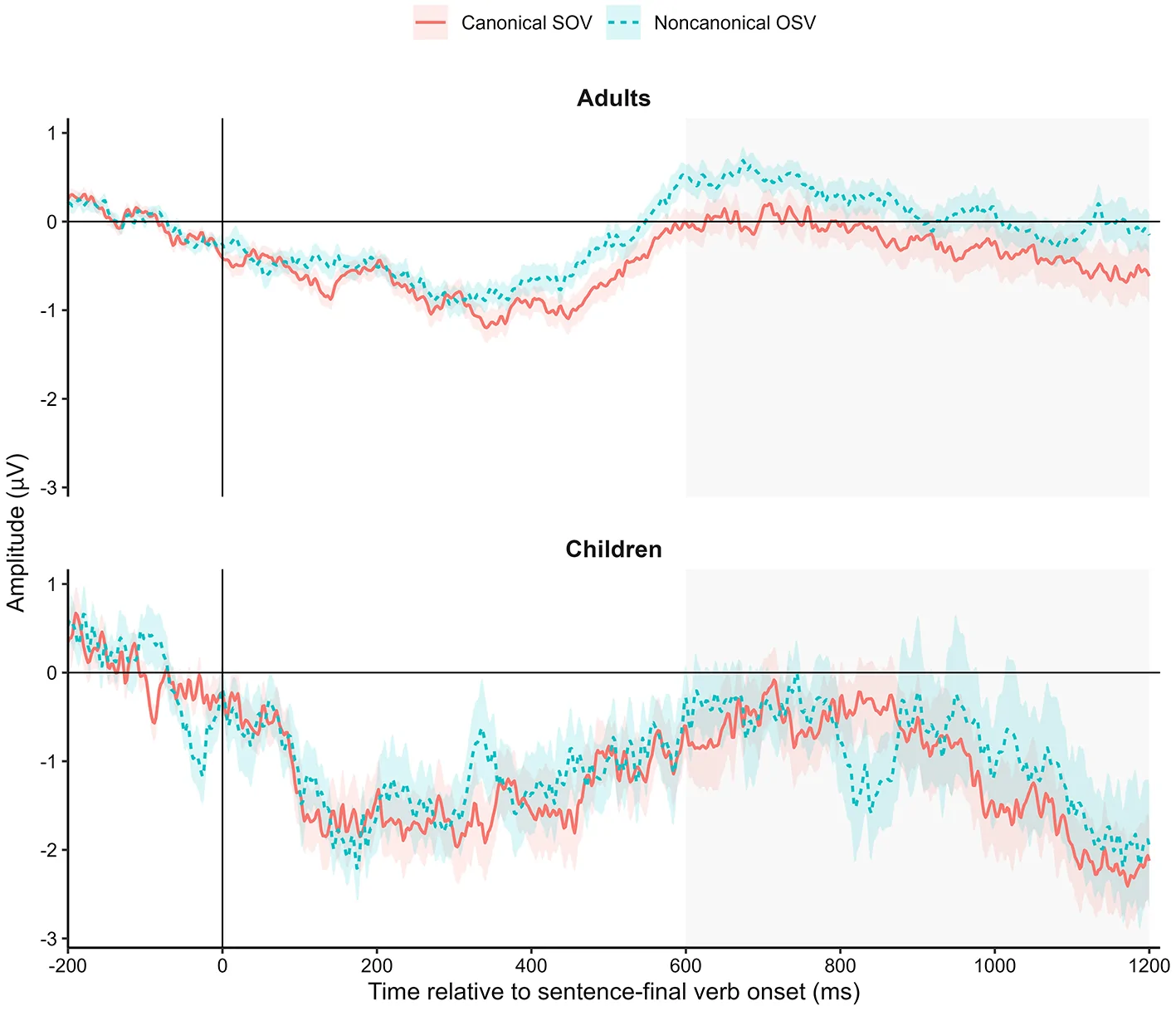
Grand-average ERP waveforms for canonical SOV and non-canonical OSV sentences in adults and children at the posterior ROI. The posterior ROI comprised P3, Pz, P4, O1, Oz, and O2. Waveforms are time-locked to sentence-final verb onset. Shaded ribbons represent ±1 standard error across participants. The gray region indicates the prespecified 600–1,200-ms analysis interval. Positive voltage is plotted upward. Adults: *n* = 67; children: *n* = 37.

**Figure 5:**
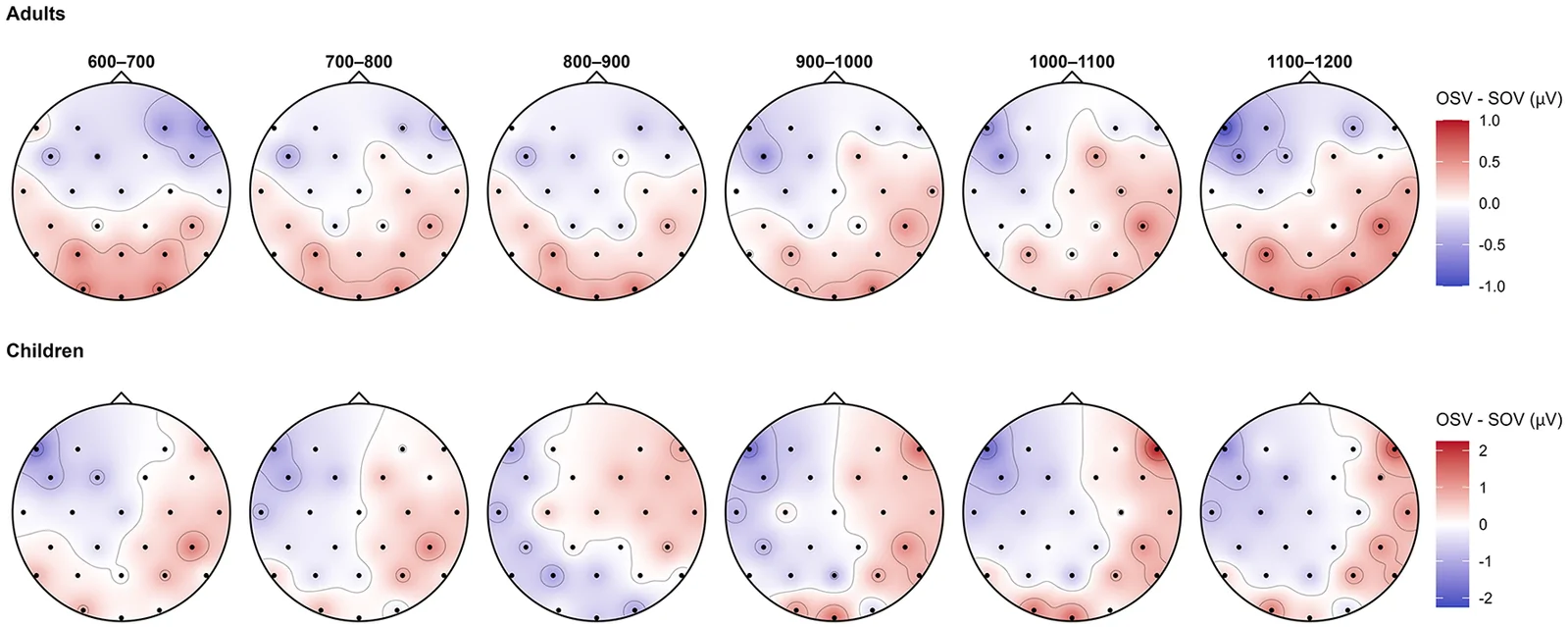
Scalp distributions of the non-canonical OSV – canonical SOV ERP amplitude difference in adults and children across the six prespecified 100-ms analysis windows. Positive values indicate greater positivity for non-canonical OSV than canonical SOV sentences. Color scales were held constant across the six windows within each group but scaled separately between groups to represent within-group spatial distributions. Scalp maps were interpolated from the 25 recorded EEG electrodes with bilateral homologs available together with the midline electrodes.

**Figure 6:**
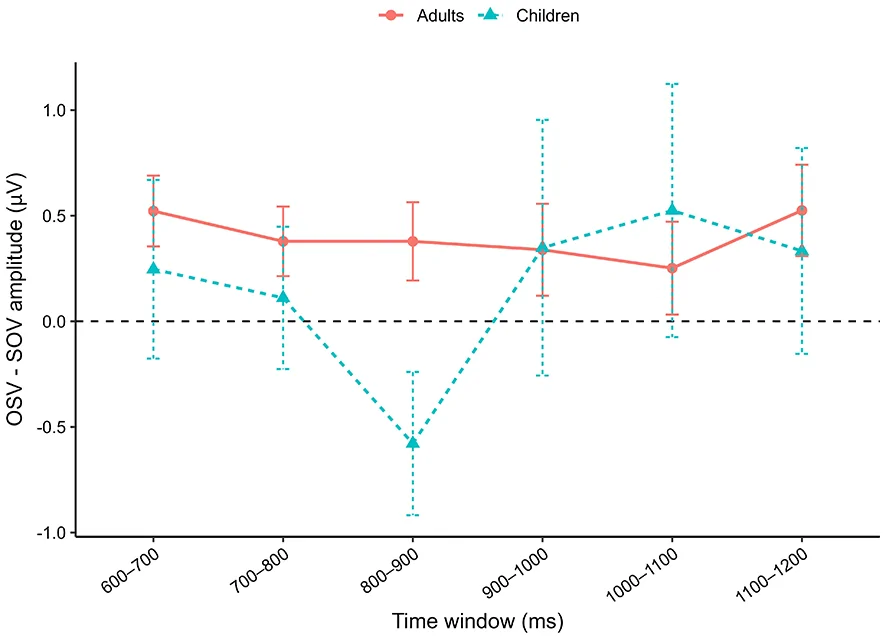
Mean non-canonical OSV – canonical SOV ERP amplitude differences across the six prespecified 100-ms analysis windows in adults and children at the posterior ROI. Positive values indicate greater positivity for non-canonical OSV than canonical SOV sentences. Error bars represent ±1 standard error of the mean. Adults: *n* = 67; children: *n* = 37. Posterior ROI = P3, Pz, P4, O1, Oz, and O2.

Descriptively, the scalp maps showed a broadly posterior positive non-canonical OSV – canonical SOV distribution in adults across several analysis windows, whereas the spatial pattern in children was less consistent across windows. The model-estimated condition contrasts and Group × Sentence Type interactions are summarized in Table 2. Full omnibus results are reported in Supplementary Table S6, within-group non-canonical OSV – canonical SOV contrasts in Supplementary Table S7, and condition-specific adult–child comparisons in Supplementary Table S8.

**Table 2:** Model-estimated non-canonical OSV – canonical SOV ERP differences and group differences in the condition effect at the posterior ROI.

Time window (ms)	Adults		Children		Adult–child difference	
	Δ*M* (*SE*)	Directional *p*FDR	Δ*M* (*SE*)	Directional *p*FDR	Δ*M* (*SE*)	Two-tailed *p*FDR
600–700	0.522 (0.166)	0.007	0.246 (0.470)	0.463	0.276 (0.499)	0.887
700–800	0.379 (0.162)	0.021	0.111 (0.407)	0.471	0.268 (0.438)	0.887
800–900	0.379 (0.182)	0.030	−0.579 (0.416)	0.916	0.957 (0.454)	0.225
900–1,000	0.339 (0.216)	0.072	0.348 (0.694)	0.463	−0.010 (0.727)	0.989
1,000–1,100	0.252 (0.218)	0.126	0.525 (0.668)	0.463	−0.273 (0.702)	0.887
1,100–1,200	0.525 (0.215)	0.021	0.333 (0.533)	0.463	0.192 (0.575)	0.887

ERP amplitudes are expressed in microvolts. ΔM represents the model-estimated non-canonical OSV minus canonical SOV difference; positive values indicate greater positivity for non-canonical OSV sentences. Within-group condition contrasts were evaluated using directional tests of OSV > SOV, with BH-FDR correction conducted separately within each group across the six time windows. Adult–child differences in ΔM correspond to the Group × Sentence Type interaction and were evaluated using two-tailed tests with BH-FDR correction across the six interactions.

The critical two-tailed Group × Sentence Type interactions did not survive BH-FDR correction in any of the six time windows (all *p*FDR ≥ 0.225; see Supplementary Table S6). The strongest uncorrected Group × Sentence Type interaction was observed at 800–900 ms, where the non-canonical OSV – canonical SOV difference was descriptively larger in adults than in children, *b* = 0.96 μV, *SE* = 0.45, *t*_(102)_ = 2.11, uncorrected *p* = 0.037, *p*FDR = 0.225. However, this interaction did not survive BH-FDR correction. Thus, the analyses did not provide corrected evidence that the OSV – SOV ERP difference varied between adults and children.

Within-group contrasts were evaluated directionally based on the hypothesized OSV > SOV positivity (Supplementary Table S7). Adults showed significant BH-FDR-corrected non-canonical OSV > canonical SOV positivities at 600–700 ms, *b* = 0.522 μV, *SE* = 0.166, *p*FDR = 0.007; 700–800 ms, *b* = 0.379 μV, *SE* = 0.162, *p*FDR = 0.021; 800–900 ms, *b* = 0.379 μV, *SE* = 0.182, *p*FDR = 0.030; and 1,100–1,200 ms, *b* = 0.525 μV, *SE* = 0.215, *p*FDR = 0.021. The contrasts at 900–1,000 and 1,000–1,100 ms did not survive correction. The adult pattern therefore showed a late non-canonical OSV > canonical SOV positivity beginning at 600–700 ms and appearing in several later posterior windows. Children showed no significant directional OSV > SOV contrast in any time window after correction (all directional *p*FDR ≥ 0.463). Descriptively, the largest child difference was negative at 800–900 ms, *b* = −0.58 μV.

Secondary two-tailed comparisons of ERP amplitude between groups (Supplementary Table S8) showed greater adult positivity for non-canonical OSV sentences at 800–900 ms, *b* = 1.25 μV, *SE* = 0.44, *t*_(102)_ = 2.84, *p*FDR = 0.021. At 1,100–1,200 ms, adults also showed greater posterior amplitudes than children for canonical SOV sentences, *b* = 1.61 μV, *SE* = 0.50, *t*_(102)_ = 3.20, *p*FDR = 0.015, and non-canonical OSV sentences, *b* = 1.80 μV, *SE* = 0.58, *t*_(102)_ = 3.10, *p*FDR = 0.015. Because the corresponding Group × Sentence Type interactions were not significant after correction, these comparisons do not provide evidence of a sentence-type-specific developmental difference in the OSV – SOV effect.

Sensitivity analyses examined whether variability in children's behavioral performance was associated with posterior ERP amplitude (Supplementary Table S9). Participant-level condition-specific accuracy showed a significant association with ERP amplitude at 600–700 ms, *F*_(1, 31)_ = 8.77, *p*FDR = 0.031, and 700–800 ms, *F*_(1, 31)_ = 7.46, *p*FDR = 0.031. Condition-specific slopes indicated that higher canonical SOV accuracy was associated with greater positivity at 600–700 ms, *b* = 0.629 μV per 10-percentage-point increase in accuracy, *SE* = 0.214, *p*FDR = 0.042, and 700–800 ms, *b* = 0.556 μV, *SE* = 0.192, *p*FDR = 0.042. The corresponding associations for non-canonical OSV accuracy did not survive correction, and no Sentence Type × Accuracy interaction was significant after correction (all *p*FDR ≥ 0.208). Thus, individual differences in behavioral accuracy were related to ERP amplitude in some early portions of the analysis interval, but there was no corrected evidence that this association differed between canonical SOV and non-canonical OSV sentences.

The child ERP condition effect was also re-evaluated after restricting the sample, first to the 35 children with available behavioral data and then to the 30 children who completed both experimental blocks (Supplementary Table S10). Neither restricted analysis yielded a significant directional non-canonical OSV > canonical SOV positivity in any time window after BH-FDR correction (children with behavioral data: all directional *p*FDR ≥ 0.678; children completing both blocks: all directional *p*FDR = 0.918). The absence of a reliably detected posterior OSV > SOV positivity in the entire child sample therefore remained unchanged when children without behavioral data or children completing only one block were excluded.

Restricting the child group to those aged 7 years or older likewise did not change the inferential ERP pattern (Supplementary Table S22). None of the directional child OSV > SOV contrasts survived BH-FDR correction across the six time windows. At 1,100–1,200 ms, the uncorrected directional test yielded *b* = 1.14 μV, *SE* = 0.67, *t*_(83)_ = 1.71, one-tailed *p* = 0.045, but this effect did not survive correction, *p*FDR = 0.268. None of the adult–child differences in the OSV – SOV effect survived correction in the age-restricted analysis (all *p*FDR ≥ 0.631).

### 3.3 Age-related differences in children's behavioral performance

The 35 children included in the behavioral analyses ranged in age from 4.33 to 9.67 years (*M* = 6.87, *SD* = 1.22). Accuracy showed a positive descriptive association with age in both sentence types. Full results from the age model are reported in Supplementary Table S11, and individual accuracy values across age are shown in Supplementary Figure S1. Each additional year of age was associated with an estimated 2.72-percentage-point increase in canonical SOV accuracy, *SE* = 2.27, 95% CI [−1.88, 7.31], *t*_(40.19)_ = 1.20, *p* = 0.239, and a 2.31-percentage-point increase in non-canonical OSV accuracy, *SE* = 2.27, 95% CI [−2.28, 6.91], *t*_(40.19)_ = 1.02, *p* = 0.316. However, these positive slopes were not statistically significant, and the omnibus association between age and accuracy was likewise not reliable, *F*_(1, 33)_ = 1.36, *p* = 0.252.

The positive age slopes were highly similar across sentence types. Accordingly, the Sentence Type × Age interaction was not significant, *F*_(1, 33)_ = 0.08, *p* = 0.777. The difference between the non-canonical OSV and canonical SOV age slopes was only −0.41 percentage points per year, *SE* = 1.43, 95% CI [−3.32, 2.51], *t*_(33)_ = −0.29, *p* = 0.777. Thus, although older children tended descriptively to achieve higher accuracy in both conditions, there was no reliable evidence that chronological age predicted overall performance or that the SOV – OSV accuracy difference changed across the sampled age range.

The quadratic-age sensitivity analysis likewise yielded no significant quadratic association between age and accuracy, Age^2^: *F*_(1, 32)_ = 0.83, *p* = 0.370, or Sentence Type × Age^2^ interaction, *F*_(1, 32)_ = 0.40, *p* = 0.529. Descriptively, the SOV – OSV accuracy difference was similar across the three age intervals: 8.23 percentage points among 4–5-year-olds, 9.63 percentage points among 6–7-year-olds, and 8.04 percentage points among 8–9-year-olds (Supplementary Table S11).

### 3.4 Age-related differences in children's ERP responses

The ERP analyses included all 37 children, whose ages ranged from 4.33 to 9.67 years (*M* = 6.83, *SD* = 1.20). To examine whether children's ERP responses to canonical SOV and non-canonical OSV sentences varied across the sampled age range, separate Sentence Type × Age models were fitted for each of the six time windows at the posterior ROI. No main effect of age survived BH-FDR correction in any time window (all *p*FDR ≥ 0.152), and no Sentence Type × Age interaction survived correction (all *p*FDR ≥ 0.828; Supplementary Table S12).

The strongest uncorrected age effect occurred at 1,100–1,200 ms, *F*_(1, 35)_ = 4.84, *p* = 0.034, *p*FDR = 0.152. Condition-specific analyses indicated that age slopes were positive for both sentence types across all time windows. At 1,100–1,200 ms, each additional year of age was associated with an estimated 1.03-μV increase in amplitude for non-canonical OSV sentences, *SE* = 0.48, *p*FDR = 0.217. The corresponding canonical SOV slope was also positive, *b* = 0.64 μV per year, *SE* = 0.37, *p*FDR = 0.217. Critically, the difference between these age slopes was not significant, *b* = 0.39 μV per year, *SE* = 0.41, *p*FDR = 0.828. Thus, older children tended descriptively to show more positive posterior ERP amplitudes, particularly in the later windows. However, these associations did not survive correction and were not selectively stronger for non-canonical OSV than canonical SOV sentences.

The quadratic-age sensitivity analysis likewise provided no corrected evidence of a non-linear developmental change in the ERP response. No Age^2^ effect survived BH-FDR correction; the smallest adjusted value occurred at 800–900 ms, *F*_(1, 34)_ = 7.85, *p* = 0.008, *p*FDR = 0.0501. Sentence Type × Age^2^ interactions were not significant in any time window (all *p*FDR ≥ 0.9629). Descriptively, non-canonical OSV – canonical SOV amplitudes varied in direction and magnitude across the 4–5-, 6–7-, and 8–9-year age intervals rather than showing a consistent age-graded pattern (Supplementary Table S13; Supplementary Figure S1).

### 3.5 Grammatical ability and children's behavioral performance beyond age

To determine whether grammatical ability accounted for variation in children's comprehension beyond chronological age, accuracy was modeled as a function of sentence type, centered age, grammar, and the interactions of sentence type with both predictors. The sentence-type effect remained significant after age and grammar were included, *F*_(1, 32)_ = 27.46, *p* < 0.001.

Grammar showed a strong positive association with accuracy beyond age, *F*_(1, 32)_ = 42.16, *p* < 0.001. A one-unit increase in the grammar composite was associated with a 16.20-percentage-point increase in canonical SOV accuracy, *SE* = 2.51, 95% CI [11.15, 21.26], *t*_(46.56)_ = 6.45, *p* < 0.001, and a 13.10-percentage-point increase in non-canonical OSV accuracy, *SE* = 2.51, 95% CI [8.04, 18.16], *t*_(46.56)_ = 5.21, *p* < 0.001 (Supplementary Table S14).

Although the estimated grammar slope was numerically larger for canonical SOV than non-canonical OSV sentences, the Sentence Type × Grammar interaction was not significant, *F*_(1, 32)_ = 1.97, *p* = 0.171. The difference between the non-canonical OSV and canonical SOV grammar slopes was −3.10 percentage points, *SE* = 2.21, 95% CI [−7.61, 1.41], *t*_(32)_ = −1.40, *p* = 0.171. Thus, stronger grammatical ability predicted higher comprehension accuracy in both sentence types, but there was no reliable evidence that this association differed between sentence types. Age was included as a covariate to isolate the association between grammatical ability and accuracy. Neither the main effect of age, *F*_(1, 32)_ = 3.67, *p* = 0.064, nor the Sentence Type × Age interaction, *F*_(1, 32)_ = 0.24, *p* = 0.629, was significant (Supplementary Table S14).

The robustness model using the overall-language composite produced the same pattern. Overall language was positively associated with accuracy beyond age, *F*_(1, 32)_ = 33.67, *p* < 0.001. A one-unit increase in the overall-language composite predicted a 15.85-percentage-point increase in canonical SOV accuracy, *SE* = 2.76, 95% CI [10.30, 21.40], *t*_(45.27)_ = 5.75, *p* < 0.001, and a 13.15-percentage-point increase in non-canonical OSV accuracy, *SE* = 2.76, 95% CI [7.60, 18.70], *t*_(45.27)_ = 4.77, *p* < 0.001 (Supplementary Table S14). The Sentence Type × Overall Language interaction was not significant, *F*_(1, 32)_ = 1.34, *p* = 0.256; slope difference: *b* = −2.70, *SE* = 2.33, 95% CI [−7.44, 2.05], *t*_(32)_ = −1.16, *p* = 0.256. Thus, higher scores on both the grammar and broader overall-language composites were associated with better comprehension, without reliable evidence that either association differed between sentence types.

FDS was examined as a sensitivity analysis in the 20 children with both FDS and behavioral data (Supplementary Table S15). FDS was positively associated with accuracy beyond age, *F*_(1, 17)_ = 13.57, *p* = 0.002. A one-unit increase in standardized FDS was associated with an 11.94-percentage-point increase in canonical SOV accuracy, *SE* = 3.35, 95% CI [4.98, 18.90], *t*_(20.79)_ = 3.57, *p* = 0.002, and an 11.43-percentage-point increase in non-canonical OSV accuracy, *SE* = 3.35, 95% CI [4.47, 18.39], *t*_(20.79)_ = 3.42, *p* = 0.003. The Sentence Type × FDS interaction was not significant, *F*_(1, 17)_ = 0.06, *p* = 0.813. In the same restricted sample, grammar remained a significant predictor after adjustment for FDS, *F*_(1, 16)_ = 15.13, *p* = 0.001, whereas the FDS effect in the grammar-adjusted model was not significant, *F*_(1, 16)_ = 1.17, *p* = 0.296. Thus, the grammar–comprehension association was robust to adjustment for verbal short-term memory.

Maternal education was also examined as a potential background correlate (Supplementary Table S16). Maternal education positively predicted the grammar composite beyond age, *b* = 0.580, *SE* = 0.155, *t* = 3.75, *p* < 0.001. However, when maternal education was added to the comprehension model, grammar remained strongly associated with accuracy, *F*_(1, 31)_ = 30.97, *p* < 0.001, whereas maternal education showed no independent association with accuracy, *F*_(1, 31)_ = 0.00, *p* = 0.992. The Sentence Type × Grammar interaction also remained non-significant, *F*_(1, 32)_ = 1.97, *p* = 0.171. These results indicate that the association between grammatical ability and comprehension was robust to adjustment for maternal educational background.

Finally, an exploratory analysis replaced the grammar composite with the standardized TODIL morphological-completion score (Supplementary Table S17). Morphological completion was positively associated with comprehension accuracy beyond age, *F*_(1, 32)_ = 27.31, *p* < 0.001. A one-unit increase in standardized morphological completion was associated with a 13.31-percentage-point increase in canonical SOV accuracy, *SE* = 2.52, 95% CI [8.23, 18.39], *t*_(44.02)_ = 5.28, *p* < 0.001, and a 10.80-percentage-point increase in non-canonical OSV accuracy, *SE* = 2.52, 95% CI [5.72, 15.88], *t*_(44.02)_ = 4.28, *p* < 0.001. The Sentence Type × Morphological Completion interaction was not significant, *F*_(1, 32)_ = 1.52, *p* = 0.226, providing no reliable evidence that the association differed between sentence types.

### 3.6 Grammatical ability and children's ERP responses beyond age

To determine whether grammatical ability explained variation in children's ERP responses beyond chronological age, ERP amplitude was modeled as a function of sentence type, centered age, grammar, and the interactions of sentence type with both predictors. Grammar did not show a significant main effect in any of the six posterior time windows after BH-FDR correction (all *p*FDR ≥ 0.591). Likewise, none of the condition-specific grammar slopes survived correction (all *p*FDR ≥ 0.404), and there was no corrected Sentence Type × Grammar interaction (all *p*FDR ≥ 0.086; Supplementary Table S18). The strongest uncorrected Sentence Type × Grammar interaction occurred at 900–1,000 ms, *F*_(1, 34)_ = 6.65, *p* = 0.014, but did not survive correction, *p*FDR = 0.086.

The overall-language robustness analysis yielded the same pattern. Overall language did not show a significant main effect in any posterior time window after BH-FDR correction (all *p*FDR ≥ 0.608), none of the condition-specific overall-language slopes survived correction (all *p*FDR ≥ 0.559), and there was no corrected Sentence Type × Overall Language interaction (all *p*FDR ≥ 0.181; Supplementary Table S19). The strongest uncorrected Sentence Type × Overall Language interaction was again observed at 900–1,000 ms, *F*_(1, 34)_ = 5.12, *p* = 0.030, *p*FDR = 0.181. Thus, neither grammatical ability nor overall language reliably predicted children's ERP amplitudes beyond age or modulated the non-canonical OSV – canonical SOV ERP difference. This contrasts with the behavioral findings, in which both language measures were strongly associated with comprehension accuracy.

Finally, the exploratory analysis using morphological completion as an alternative predictor likewise provided no corrected evidence of an association with children's posterior ERP responses (Supplementary Table S17). No main effect of morphological completion survived BH-FDR correction in any time window (all *p*FDR = 0.711), none of the condition-specific morphological-completion slopes survived correction (all *p*FDR ≥ 0.554), and no Sentence Type × Morphological Completion interaction survived correction (all *p*FDR ≥ 0.297). Thus, although morphological completion was strongly associated with behavioral comprehension accuracy, it did not reliably predict posterior ERP amplitude or modulation of the non-canonical OSV – canonical SOV ERP difference.

## 4 Discussion

The present study examined the behavioral and electrophysiological consequences of canonical SOV and non-canonical OSV word order in Turkish-speaking adults and children, as well as the extent to which variation within childhood was associated with chronological age and grammatical ability. Non-canonical OSV sentences were comprehended less accurately than canonical SOV sentences in both groups, and this SOV–OSV accuracy difference was significantly larger in children. At the neural level, adults showed the predicted verb-locked non-canonical OSV > canonical SOV late positivity over the posterior ROI, consistent with increased processing demands associated with the non-canonical configuration. Children, in contrast, showed no reliable corresponding positivity after correction for multiple comparisons. Importantly, however, the critical Group × Sentence Type interactions were also not significant after correction, and the present results therefore do not establish that the SOV–OSV ERP difference was reliably different in magnitude between adults and children. Within the child group, chronological age was not reliably associated with comprehension accuracy, the behavioral SOV–OSV difference, or the SOV–OSV ERP difference across the sampled age range. Grammatical ability, by contrast, strongly predicted comprehension accuracy after age was taken into account, a pattern that was replicated using overall language ability, although neither measure was specifically associated with the additional behavioral disadvantage for non-canonical OSV or reliably related to the ERP response. Together, these findings indicate that grammatical non-canonical OSV word order is associated with additional behavioral difficulty despite the availability of informative case cues, while the corresponding late neural response was reliably detected in adults but not in children, without evidence that the magnitude of the ERP effect differed between groups after correction for multiple comparisons.

### 4.1 Behavioral consequences of non-canonical OSV word order

The behavioral findings provide clear evidence that non-canonical OSV word order was associated with additional comprehension difficulty under the conditions of the present experiment. Comprehension accuracy was lower for non-canonical OSV than canonical SOV sentences in both adults and children, with an SOV–OSV accuracy difference of 2.77 percentage points in adults and 8.91 percentage points in children. Critically, this difference was significantly larger in children. This pattern extends previous evidence for a canonical-order advantage in Turkish adults. Ergin and Stromswold (2023) reported greater processing difficulty for non-canonical word orders, while (Bulut and Günhan Senol 2026), using closely comparable reversible SOV and OSV sentences, found lower comprehension accuracy and longer response latencies for non-canonical OSV sentences, together with longer reading times at both the first noun phrase and the sentence-final verb. The present findings add a developmental comparison using the same controlled sentence contrast, showing that the behavioral disadvantage for non-canonical OSV is substantially greater in children than in adults.

The canonical and non-canonical versions contained exactly the same lexical items and were matched in event plausibility, eliminating a straightforward between-condition lexical-content or lexical-frequency confound. This control does not, however, establish that every item was equally accessible to the youngest children. Developmental familiarity and visual identifiability were explicit criteria during stimulus preparation, but the retrospective AoA norms obtained later indicated that several of the profession-denoting nouns had later estimated acquisition ages. Importantly, the larger behavioral SOV–OSV difference in children was also evident when the analysis was restricted to those aged 7 years or older, who were at or above the median retrospective AoA estimate for every experimental lexical item. Lexical accessibility may therefore have contributed to overall difficulty among the youngest children, but it is unlikely to fully explain the larger SOV–OSV behavioral difference.

These within-item controls also do not equate the broader linguistic experience associated with the two structures. SOV is strongly dominant over OSV in Turkish corpus data (Türk et al., 2022), and such distributional asymmetry plausibly contributes to stronger expectations for canonical order. At the same time, corpus-level frequency is not equivalent to an individual speaker's experience: in adult native speakers of English, print exposure and education are associated with individual differences in grammar, vocabulary, and collocational knowledge (Dabrowska, 2018), whereas in adult native speakers of Turkish, print exposure was associated with how speakers extended an inflectional pattern to novel verbs (Gedik, 2024). Structural frequency is therefore likely to be one relevant source of processing expectation rather than a complete explanation of the present SOV–OSV difference. Word order is also closely linked to discourse and information-structural properties in Turkish (Erguvanli Taylan, 1984; Işsever, 2003; Türk et al., 2022), and the present sentences were presented without a preceding context specifically licensing OSV order.

The particularly large SOV–OSV accuracy difference in children should not, however, be interpreted as evidence that children are unable to identify or use morphological case information. Turkish-speaking children acquire case morphology relatively early (Ketrez and Aksu-Koç, 2009) and use case marking in thematic-role assignment from an early age (Slobin and Bever, 1982). More importantly for the present materials, eye-tracking studies show that children can exploit case information incrementally before the sentence-final verb. Özge et al. (2019) demonstrated predictive use of case marking in Turkish-speaking children from approximately four years of age, and Tiryakiol et al. (2026) likewise found rapid online sensitivity to overt accusative marking in non-canonical sentences. The key distinction, therefore, is between the availability of a cue and how that cue is weighted and integrated with other information during sentence processing. Early access to accusative marking can permit identification of thematic roles without making a less expected argument order as easy to process as the dominant SOV configuration. Previous eye-tracking work using the present sentence materials is consistent with this distinction: children exploited the case-marked information online but showed a later time course for the non-canonical OSV interpretation than for canonical SOV, whereas adults showed earlier target preferences in both conditions (Işleyen, 2022; Yaman, 2022). Because these findings came from separate studies and were not directly compared statistically, they should be interpreted cautiously. Nevertheless, they converge with the present behavioral results in showing that successful use of case marking and an additional processing disadvantage for non-canonical order can coexist. A similar distinction has been observed in German, where children who could use case marking successfully in sentence interpretation nevertheless continued to show additional processing demands for object-initial structures (Schipke et al., 2012).

The delayed-response format of the present task also means that successful performance required children to retain the interpreted sentence until the pictures appeared, raising the possibility that verbal short-term or working-memory resources contributed to comprehension performance (Boyle et al., 2013; Montgomery et al., 2008). The forward-digit-span sensitivity analysis supports a contribution of verbal short-term memory to overall comprehension performance but does not provide evidence that it specifically explains the larger OSV disadvantage. In the restricted sample with available forward digit span (*n* = 20), stronger verbal short-term memory predicted higher accuracy in both sentence types, but the Sentence Type × Forward Digit Span interaction was not significant. Moreover, grammatical ability remained associated with comprehension after adjustment for forward digit span, whereas forward digit span was no longer independently significant once grammar was included. Given the restricted sample, these findings should be interpreted cautiously, but they do not support an account in which the non-canonical disadvantage is primarily a consequence of verbal short-term-memory limitations.

At the same time, the present findings should not be taken to imply an invariant processing penalty associated with OSV order. Non-canonical word order in Turkish is closely tied to discourse and information structure, and non-canonical orders can be more felicitous when supported by an appropriate context (Erguvanli Taylan, 1984; Işsever, 2003). Cross-linguistic evidence likewise demonstrates that processing costs associated with non-canonical word order depend on discourse conditions. In Japanese, for example, Yano and Koizumi (2018) found that the processing consequences of OSV word order were reduced when the discourse context appropriately licensed the non-canonical structure. The present sentences were presented without a preceding discourse context specifically designed to provide discourse support for OSV order. The behavioral disadvantage for OSV word order should therefore be interpreted as a cost associated with processing the non-canonical configuration under the experimental conditions used here, rather than as an intrinsic or context-independent property of OSV word order.

### 4.2 Adult late positivity during sentence-final integration

The adult ERP results provide converging evidence that the additional processing demands associated with non-canonical OSV word order are expressed during sentence-final processing. Non-canonical OSV sentences elicited greater positivity than canonical SOV sentences at the posterior ROI, with significant BH-FDR-corrected effects beginning at 600–700 ms and appearing across several subsequent windows. The timing and posterior emphasis of this late positivity make the P600/syntactic positive shift (SPS) literature particularly relevant to its interpretation. Because both sentence types were grammatical and morphologically disambiguated, the effect is particularly informative about late processing demands arising in a grammatical canonical–non-canonical contrast rather than about the detection of a grammatical violation.

The P600/SPS was initially identified in studies of syntactic anomaly and parsing difficulty (Hagoort et al., 1993; Osterhout and Holcomb, 1992), but has subsequently been linked to a broader range of operations involved in constructing and revising sentence structure. Hagoort and Brown (2000), for example, distinguished an earlier aspect of the P600/SPS associated with structural integration complexity from a later, more posterior phase associated with unsuccessful parsing or reanalysis, and demonstrated closely comparable P600/SPS responses during spoken and written sentence comprehension. Importantly for the present study, subsequent work established that a late positivity does not require an outright grammatical violation. Kaan et al. (2000) elicited a P600 in grammatical, non-garden-path sentences when syntactic integration was relatively demanding, supporting the proposal that the component is sensitive to integration difficulty more generally.

A particularly relevant formulation is provided by Hagoort's (2003) Unification Model. On this account, lexical items activate stored syntactic frames that must be dynamically combined, or unified, into a structural representation spanning the utterance. Hagoort proposed that the P600/SPS is related to the time required to establish sufficiently strong unification links, with greater competition or structural complexity increasing these demands. This conception was subsequently embedded within the broader Memory, Unification, and Control framework, in which sentence comprehension requires information retrieved from linguistic memory to be combined into larger syntactic and semantic representations (Hagoort, 2005, 2013). From this perspective, the present non-canonical OSV > canonical SOV positivity is compatible with greater demands on unifying the incoming verb with the previously encountered case-marked arguments when those arguments occur in a non-canonical configuration. This interpretation is also compatible with broader accounts in which the P600 reflects the construction or updating of the unfolding sentence-level representation (Brouwer et al., 2012, 2017). The present data do not distinguish among these accounts, but they converge on the idea that late positive activity can reflect increased combinatorial or integrative demands even when the resulting sentence is fully grammatical.

Against this theoretical background, the location of the effect at the sentence-final verb is particularly informative. In Turkish, the preceding case-marked noun phrases already provide information about their grammatical and thematic roles before the verb is encountered (Özge et al., 2019). The verb nevertheless supplies the predicate and its argument-structure information, providing a point at which the preceding arguments can be incorporated into a complete event representation. The present ERP findings converge with previous behavioral evidence from Turkish using reversible SOV and OSV sentences. (Bulut and Günhan Senol 2026) observed longer reading times for non-canonical OSV sentences not only at the initial noun phrase but also at the sentence-final verb. Moreover, individual differences in visual statistical learning modulated the SOV–OSV reading-time difference at the verb but not at the first noun phrase or intervening regions. Thus, the verb was not the first point at which the two structures differed in processing difficulty, but it was a later locus at which the SOV–OSV difference was also related to individual variation. Although the individual-difference measures in the present study concerned age and language-related abilities rather than statistical learning, this finding further supports the verb as an informative position for examining variability in the processing consequences of non-canonical order.

Particularly relevant electrophysiological evidence comes from Japanese, another verb-final language permitting SOV–OSV scrambling. Yano and Koizumi (2018) reported a larger P600 for OSV than SOV at the verb under a discourse condition that did not facilitate the scrambled order and related this effect to thematic-role assignment and the integration of the verb with its arguments. The context sensitivity of that effect is also important for interpreting the present findings. The adult positivity observed here should not be treated as a pure electrophysiological measure of syntactic movement or scrambling *per se*: the SOV and OSV conditions differed not only in argument order but also in distributional frequency, the position of accusative marking, and information-structural fit in the absence of a licensing discourse context. The present effect is therefore most conservatively interpreted as reflecting increased late processing demands associated with the grammatical non-canonical OSV configuration under the conditions of the present experiment. Together with previous behavioral and electrophysiological findings, the present results suggest that the sentence-final verb constitutes an important locus of processing difficulty for grammatical non-canonical OSV sentences.

### 4.3 Children's behavioral and neural responses to non-canonical OSV word order

The child results revealed a marked asymmetry between behavioral and electrophysiological responses to the SOV–OSV contrast. Behaviorally, children showed a substantially larger reduction in comprehension accuracy for non-canonical OSV than canonical SOV sentences than adults. At the neural level, however, children did not show a reliable non-canonical OSV > canonical SOV late positivity in any of the prespecified posterior-ROI time-window tests after correction for multiple comparisons. Thus, the pronounced behavioral disadvantage for non-canonical OSV in children was not accompanied by a reliably detected late posterior positivity of the form observed in adults. This contrasts descriptively with the adult pattern, in which significant late positivities were detected across several time windows at the same posterior ROI.

This behavioral–electrophysiological dissociation is informative in light of the developmental ERP literature. Neural sensitivity to sentence structure emerges early, but the timing, magnitude, and scalp distribution of late positive responses continue to vary across childhood and across linguistic manipulations (Atchley et al., 2006; Hahne et al., 2004; Schneider and Maguire, 2019). Particularly relevant evidence comes from studies in which children must use morphosyntactic cues to interpret grammatical argument structures. In German, Schipke et al. (2012) found that 6-year-old children successfully used morphological case for thematic-role assignment but nevertheless showed additional electrophysiological processing demands for object-initial sentences that were not observed in adults. Similarly, Kruchinina et al. (2022) found ERP sensitivity to the relevant inflectional cues during the comprehension of reversible active and passive sentences in Russian-speaking children aged four to six, whereas a P600 was observed only in the 6-year-old group. Related evidence from Basque likewise indicates that case information can be processed successfully while its neural expression differs across development: Isasi-Isasmendi et al. (2025) reported both shared and age-dependent electrophysiological responses to case marking in adults and 6-year-old children. Together, these findings show that successful use of morphosyntactic information need not be accompanied by an adult-identical neural response and that developmental differences may concern when, where, or how strongly such information is expressed electrophysiologically.

The present child findings fit within this broader developmental picture, but they do not identify which developmental explanation accounts for the absence of a reliably detected OSV > SOV positivity. A smaller condition effect, greater inter-individual variability, differences in its temporal course or scalp distribution, or limited statistical power in the child sample could all reduce the likelihood of detecting the same posterior pattern observed in adults. The present analyses cannot distinguish among these possibilities. Importantly, the absence of a significant within-group effect in children and the presence of such an effect in adults do not by themselves demonstrate that the SOV–OSV ERP difference varied between the groups. The critical Group × Sentence Type interactions did not survive correction for multiple comparisons. The most appropriate conclusion is therefore that the predicted late positivity was reliably detected in adults but not in children, while the present data provide insufficient evidence that the magnitude of the SOV–OSV ERP difference itself differed reliably between adults and children. Secondary analyses did reveal some group differences in ERP amplitude, including greater adult posterior positivity for non-canonical OSV sentences at 800–900 ms and greater adult positivity for both sentence types at 1,100–1,200 ms. However, because these effects were not accompanied by reliable Group × Sentence Type interactions, they do not provide evidence for an OSV-specific developmental difference in the condition effect.

Interpretation of the child ERP pattern is also qualified by the relationship between neural and behavioral performance. The primary ERP analysis retained all artifact-free trials irrespective of subsequent response accuracy because the ERP was intended to characterize neural processing elicited by the sentence itself, whereas the behavioral response was a later outcome obtained only after the post-sentence interval and presentation of the response pictures. Restricting the averages to correct-response trials would therefore condition the ERP analysis on a subsequent behavioral outcome and, particularly in children, would also substantially and unequally reduce the number of usable trials across sentence types. This choice nevertheless has an important interpretive consequence: because children's behavioral accuracy was substantially lower than adults', particularly for non-canonical OSV sentences, their ERP averages may combine trials that ultimately resulted in successful and unsuccessful thematic interpretations. Participant-level sensitivity analyses showed that higher condition-specific accuracy was associated with greater posterior positivity in the earlier portions of the analyzed interval, although the Sentence Type × Accuracy interactions did not survive correction for multiple comparisons. Thus, behavioral performance was related to ERP amplitude at the participant level in some time windows, but there was no reliable evidence that this relationship differed between canonical SOV and non-canonical OSV sentences. Because the ERP averages were not separated according to subsequent response accuracy, the present data do not establish whether the late neural response differed between trials that ultimately resulted in correct versus incorrect sentence interpretations.

The within-child age analyses provided a more nuanced picture. Behaviorally, older children tended to achieve higher comprehension accuracy in both sentence types: the estimated slopes corresponded to a 2.72-percentage-point increase in canonical accuracy and a 2.31-percentage-point increase in non-canonical OSV accuracy per year of age. These positive associations did not reach statistical significance, however, and the age slopes were highly similar across the two sentence types. Thus, while the data were descriptively consistent with improving sentence comprehension across the sampled age range, there was no evidence that the additional behavioral disadvantage for non-canonical OSV changed systematically with age. The additional analyses examining the broad age range yielded a similar picture. A quadratic age term did not reliably predict accuracy or interact with Sentence Type, and the descriptive SOV–OSV accuracy differences were comparable among 4–5-year-olds (8.23 percentage points), 6–7-year-olds (9.63 percentage points), and 8–9-year-olds (8.04 percentage points). Thus, the behavioral data showed neither a reliable linear or quadratic change in the SOV–OSV difference nor an obvious descriptive age interval at which the disadvantage for OSV markedly changed.

A broadly similar pattern emerged in the ERP data. ERP amplitudes tended descriptively to become more positive with age, particularly in later posterior windows, but these associations did not survive correction for multiple comparisons and were not selectively stronger for non-canonical OSV sentences. Quadratic-age analyses likewise yielded no corrected Sentence Type × Age^2^ interaction, and descriptive OSV – SOV amplitudes varied in both direction and magnitude across the three age intervals rather than exhibiting a consistent age-graded pattern. Accordingly, the present findings do not support a simple linear or quadratic age-related change in the additional behavioral or late neural response associated with non-canonical OSV word order between 4.33 and 9.67 years.

These age results should not be interpreted as evidence that the processing of non-canonical word order is developmentally stable throughout this age span. The behavioral and ERP age analyses included only 35 and 37 children, respectively, and the distribution across the descriptive age intervals was uneven, with relatively few children in the oldest group. The sample therefore provides limited sensitivity for detecting developmental interactions or more complex changes in processing strategy. Moreover, the data are cross-sectional and characterize age-related variation between children rather than developmental change within individuals. The present findings are consequently best understood as showing that no systematic linear or quadratic developmental pattern was detected within this sample, while leaving open the possibility of subtler, more variable, or differently timed developmental changes.

### 4.4 Grammatical ability and sentence comprehension in children

Whereas chronological age showed only descriptive associations with children's comprehension performance, grammatical ability was strongly related to accuracy. After age was included in the model, higher grammatical ability predicted substantially better comprehension of both canonical SOV and non-canonical OSV sentences. A one-unit increase in the grammar composite was associated with a 16.20-percentage-point increase in canonical SOV accuracy and a 13.10-percentage-point increase in non-canonical OSV accuracy. Importantly, however, the association did not differ significantly between sentence types. Thus, stronger grammatical ability was associated with more successful sentence interpretation generally, rather than specifically with a smaller behavioral disadvantage for non-canonical OSV. Because both conditions required children to recover the thematic relations of reversible, case-marked sentences, this general association is consistent with grammatical ability contributing to successful sentence interpretation across the two word orders. It does not, however, indicate that stronger grammatical ability selectively reduced the additional difficulty associated with non-canonical OSV. The exploratory analysis of morphological completion, one component of the grammar composite, showed the same general pattern: higher scores predicted better accuracy in both sentence types, without a reliable Sentence Type interaction.

The importance of concurrent grammatical ability is consistent with evidence that children of similar chronological ages can differ substantially in the linguistic knowledge and processing resources they bring to sentence comprehension. Knoll et al. (2012), for example, found that preschool children's neural responses to canonical and non-canonical case-marked German sentences covaried with grammatical knowledge and argued that differences in processing strategy were more closely related to individual grammatical proficiency than to age alone. Related evidence from Turkish also indicates that individual language abilities contribute to children's use of morphosyntactic cues during online comprehension. Özkan et al. (2022) found that early productive vocabulary, current language-production ability, and episodic-buffer performance were associated with children's ability to use case marking predictively, whereas receptive grammar measured at the time of testing was not. The difference from the present finding may reflect, among other methodological differences, the measures and processing outcomes examined: Özkan et al. focused on predictive use of case information during online comprehension, whereas the present grammar composite combined syntactic understanding, sentence imitation, and morphological completion and was related to the eventual interpretation of the complete sentence. The present findings therefore complement this earlier work by showing that variation in current grammatical ability is strongly associated with children's success in interpreting reversible case-marked sentences.

The robustness analyses also help delimit the specificity of this relationship. The broader overall-language composite produced essentially the same behavioral pattern, strongly predicting accuracy in both sentence types without a reliable Sentence Type × Overall Language interaction. Thus, although grammatical ability was the theoretically motivated primary predictor, successful comprehension was also associated with children's concurrent language ability more broadly. Verbal short-term memory contributed to performance as well: in the smaller subsample with forward-digit-span data, higher forward digit span predicted greater accuracy in both sentence types. Importantly, however, grammatical ability remained strongly associated with comprehension after adjustment for forward digit span, whereas forward digit span was no longer independently significant after grammar was included. Given the restricted sample, this result should be interpreted cautiously, but it supports the robustness of the grammar–comprehension association to adjustment for forward digit span.

Maternal educational background may also be relevant to individual differences in children's grammatical ability. A broader literature has linked maternal education and family socioeconomic background to children's language development, including measures of spontaneous language such as mean length of utterance (Dollaghan et al., 1999), receptive and expressive language outcomes (Reilly et al., 2010), and grammatical development during the preschool years (Weinert and Ebert, 2013). Differences in the home language and literacy environment and in parent–child interaction have been identified as potential pathways through which educational and socioeconomic background may be related to children's language development (Ebert et al., 2020; Sengül-Inal et al., 2025). Consistent with this literature, maternal education significantly predicted the grammar composite beyond chronological age in the present sample, indicating that children whose mothers had higher educational attainment tended to show stronger grammatical ability. When maternal education was subsequently included alongside grammatical ability in the comprehension model, grammatical ability remained strongly associated with accuracy, whereas maternal education did not explain additional variation in comprehension performance. Thus, maternal educational background was related to children's grammatical ability but did not account for the grammar–comprehension association. Maternal education nevertheless represents only one aspect of family background and should not be treated as a comprehensive measure of socioeconomic status, and the present cross-sectional analyses do not establish a causal pathway linking maternal education, grammatical ability, and sentence comprehension.

A different pattern emerged for the ERP measures. Neither grammatical ability nor overall language reliably predicted late ERP amplitude after correction for multiple comparisons, and neither measure reliably modulated the SOV–OSV ERP difference. The exploratory morphological-completion analysis likewise showed no corrected association with posterior ERP amplitude and no reliable modulation of the SOV–OSV difference. This dissociation from the behavioral results is not necessarily unexpected. Behavioral accuracy reflects the outcome of processing the sentence as a whole, whereas the present ERP measures capture a temporally circumscribed neural response elicited by the sentence-final verb. Moreover, the magnitude of the late positivity observed here should not be treated as a direct quantitative index of grammatical ability. Previous studies have reported associations between language proficiency and P600 characteristics, particularly in paradigms involving morphosyntactic violations (e.g., Pakulak and Neville, 2010; Wray and Weber-Fox, 2013), but the present manipulation contrasted two fully grammatical sentence structures. In such a contrast, greater grammatical or broader language ability could in principle affect multiple aspects of processing without producing a monotonic increase or decrease in late positivity. The present results therefore suggest that concurrent grammatical and broader language abilities are strongly related to children's success in interpreting these sentences, but were not reliably associated with posterior verb-locked ERP amplitude or with the SOV–OSV ERP difference.

### 4.5 Limitations

Several features of the present design delimit the interpretation of the findings and point to useful directions for future work. The canonical SOV–non-canonical OSV contrast does not isolate a single source of processing difficulty. Moving from SOV to OSV changes not only argument order but also the position at which accusative case marking occurs, and the two orders differ markedly in distributional frequency and in their information-structural fit in the absence of a licensing discourse context. The present experiment was not designed to separate these factors. Accordingly, the observed behavioral and ERP differences should be interpreted as consequences of processing grammatical non-canonical OSV sentences under the present experimental conditions, rather than as a specific effect of syntactic movement or scrambling itself. Future studies manipulating discourse support or otherwise separating the contributions of word order, case position, and distributional experience would help determine which properties contribute most strongly to the observed processing differences.

The electrophysiological analyses were restricted to activity time-locked to the sentence-final verb and focused on the 600–1,200-ms interval, because the primary electrophysiological question addressed here concerned late sentence-final processing of the canonical SOV and non-canonical OSV configurations. The present data therefore do not address the earlier stages at which case information first becomes available. This distinction is particularly relevant in Turkish, where previous eye-tracking work has shown that both children and adults can exploit case marking incrementally before the verb (Özge et al., 2019; Tiryakiol et al., 2026), including in studies using the present sentence materials (Işleyen, 2022; Yaman, 2022). A related limitation concerns the −200 to 0 ms baseline interval. Because this interval immediately preceded verb onset and therefore corresponded to the final portion of the second noun phrase or the transition to the verb, it cannot be assumed to have been electrophysiologically neutral with respect to sentence type. Condition-related activity may already have been present before verb onset, and baseline correction could therefore have influenced the estimated post-verbal differences. Future ERP work examining responses time-locked to the first and second noun phrases in addition to the final verb could more directly characterize how early case-based interpretation relates to the later integrative response observed here.

The task structure also places some constraints on the interpretation of behavioral performance. Response pictures appeared only after sentence offset and a 1,000-ms post-sentence interval, requiring participants to retain the interpreted sentence until the response alternatives became available. This retention requirement may have contributed to performance, particularly among the younger children. Consistent with this possibility, the forward-digit-span sensitivity analysis showed that verbal short-term memory was positively associated with comprehension accuracy beyond age in the restricted sample with available data. Importantly, however, this association was comparable for canonical SOV and non-canonical OSV sentences, with no Sentence Type × Forward Digit Span interaction. Thus, verbal short-term memory appears to have contributed to overall task performance but does not provide evidence that the greater difficulty with non-canonical OSV sentences was specifically driven by the retention demands of the task. In addition, the same complementary picture pairs recurred across the two experimental blocks, with their target and alternative roles reversed across the corresponding sentence versions. Repeated exposure to the pictures could potentially have influenced later responses, including through carryover from an earlier encounter with the same picture pair, although this repetition was systematic across the experimental design rather than specific to one sentence type.

Developmental familiarity and visual identifiability were explicit criteria during stimulus preparation, but lexical knowledge of each item and recognition of each picture were not independently verified in the original child participants. The retrospective subjective AoA ratings obtained subsequently indicated that several profession-denoting nouns had median estimated acquisition ages above 4 years, making uniform lexical familiarity less certain among the youngest children. Similarly, although black-and-white line drawings were selected to standardize the visual displays and reduce uncontrolled variation in surface features, their format may have affected accessibility for some participants. Reis et al. (2006) found that illiterate older adults benefited more than literate controls from color when naming two-dimensional representations of common objects, with group differences evident for black-and-white but not colored stimuli. Although their participants and pictorial materials differed from those used here, their findings indicate that the accessibility of two-dimensional object representations can vary with literacy-related experience and the availability of color information. The use of the same lexical items and picture pairs across sentence-type conditions therefore does not eliminate the possibility that lexical or pictorial difficulty imposed greater costs when processing the more demanding OSV configuration. The larger SOV–OSV difference remained evident when the behavioral analysis was restricted to children aged 7 years or older. Nevertheless, this sensitivity analysis cannot establish item-specific lexical knowledge or picture recognition in the original participants, particularly the youngest children.

A further qualification concerns the inclusion of all artifact-free ERP trials irrespective of subsequent behavioral response accuracy. The ERP analysis targeted neural processing elicited by the sentence itself, whereas the behavioral response was obtained later, after the post-sentence interval and presentation of the response pictures. Restricting averages to correct-response trials would additionally have reduced the number of usable trials, particularly in children and especially for non-canonical OSV sentences, for which accuracy was lower. Nevertheless, because children's accuracy was considerably lower than adults', especially for non-canonical OSV sentences, the child ERP averages may combine trials that ultimately led to correct and incorrect sentence interpretations. Participant-level sensitivity analyses showed that behavioral accuracy was associated with posterior ERP amplitude at 600–700 and 700–800 ms, although no Sentence Type × Accuracy interaction survived correction for multiple comparisons. Thus, behavioral performance was related to ERP amplitude in some portions of the analysis interval, but there was no reliable evidence that this relationship differed between canonical SOV and non-canonical OSV sentences. The primary child ERP result was also unchanged when the analyses were restricted to the 35 children with available behavioral data and, separately, to the 30 children who completed both experimental blocks. These sensitivity analyses indicate that the absence of a reliably detected child OSV > SOV positivity was not dependent on the inclusion of children without behavioral data or those who completed only one block. They nevertheless do not determine whether the neural response differed between individual trials that ultimately resulted in correct versus incorrect sentence interpretations.

Finally, the age analyses were cross-sectional. They characterize associations between chronological age and performance across the sampled children rather than developmental change within individuals. The behavioral and ERP analyses included relatively modest samples of 35 and 37 children, respectively, spanning 4.33–9.67 years, with unequal numbers across the descriptive age intervals. Additional analyses examined both linear and quadratic age relationships, and descriptive comparisons were provided for 4–5-, 6–7-, and 8–9-year-olds; these analyses did not reveal a systematic age-related change in the SOV–OSV difference. Nevertheless, the sample provides limited sensitivity for detecting subtler non-linear changes, developmental transitions, or heterogeneous processing trajectories. The absence of a reliable age-related association should therefore not be interpreted as evidence that these responses remain stable over childhood. Longitudinal data or larger samples providing denser coverage across childhood would allow the developmental trajectory of canonical and non-canonical sentence processing to be characterized more directly.

## 5 Conclusion

The present study shows that grammatical non-canonical OSV word order is associated with lower comprehension accuracy than canonical SOV order in both Turkish-speaking adults and children despite morphologically explicit information about thematic roles, with a substantially larger behavioral SOV–OSV difference in children. Adults additionally showed a verb-locked late posterior positivity for non-canonical OSV relative to canonical SOV sentences, consistent with increased sentence-final processing demands associated with the non-canonical configuration under the present experimental conditions. A corresponding effect was not reliably detected in children, although the direct adult–child difference in the SOV–OSV ERP effect did not survive correction for multiple comparisons; the present data therefore do not establish that the magnitude of this neural effect differed between groups. Within childhood, stronger concurrent grammatical ability, and similarly broader language ability, was robustly associated with more successful comprehension of both canonical SOV and non-canonical OSV sentences, without selectively reducing the OSV disadvantage, whereas chronological age showed no reliable linear or quadratic association with the SOV–OSV behavioral or ERP difference across the sampled age range. Overall, the findings indicate that case marking permits interpretation of both word orders but does not eliminate the additional behavioral difficulty associated with non-canonical OSV, particularly in children. At the neural level, a late posterior OSV > SOV positivity was reliably detected in adults but not in children, while the present evidence is insufficient to conclude that the size of this ERP effect differs developmentally.

## Data Availability

The datasets and analysis code used in this study can be found at Open Science Framework: https://doi.org/10.17605/OSF.IO/4NDP7.
