# Supplementary material for: Processing canonical and non-canonical word orders in Turkish: behavioral and ERP evidence from adults and children
Source: Front Hum Neurosci. 2026 Oct 7;20:1968429. doi: 10.3389/fnhum.2026.1968429 (PMC13683596; doi:10.3389/fnhum.2026.1968429)
Supplement: Supplementary file 1 [file Table_1.docx]

**Supplementary Material**

**for**

**Processing canonical and noncanonical word orders in Turkish: Behavioral and ERP evidence from adults and children**

The supplementary material provides additional information on the experimental materials, participant characteristics, primary statistical analyses, and sensitivity and robustness analyses reported in the manuscript. It includes the complete set of canonical SOV and noncanonical OSV sentence materials; item-level lexical-frequency and age-of-acquisition information for the experimental nouns and verbs; descriptive information on child language, memory, and parental-education measures; detailed behavioral and posterior ERP model results; analyses of age-related variation, condition-specific behavioral accuracy, and restricted child subsamples; age-restricted behavioral and ERP sensitivity analyses addressing developmental lexical accessibility; and supplementary analyses examining grammar, overall language, forward digit span, maternal education, and morphological completion. Supplementary Figure S1 presents individual behavioral and posterior ERP values across child age as descriptive visualizations.

**Supplementary Table S1.** Complete list of experimental sentence materials.

| **Set** | **Canonical SOV (Turkish)** | **English translation** | **Noncanonical OSV (Turkish)** | **English translation** |
| --- | --- | --- | --- | --- |
| 1 | Polis dedektifi bağlıyor. | The police officer is tying the detective. | Polisi dedektif bağlıyor. | The detective is tying the police officer. |
| 2 | Polis palyaçoyu sürüklüyor. | The police officer is dragging the clown. | Polisi palyaço sürüklüyor. | The clown is dragging the police officer. |
| 3 | Polis doktoru boyuyor. | The police officer is painting the doctor. | Polisi doktor boyuyor. | The doctor is painting the police officer. |
| 4 | Polis astronotu ıslatıyor. | The police officer is wetting the astronaut. | Polisi astronot ıslatıyor. | The astronaut is wetting the police officer. |
| 5 | Dedektif palyaçoyu boyuyor. | The detective is painting the clown. | Dedektifi palyaço boyuyor. | The clown is painting the detective. |
| 6 | Dedektif doktoru ıslatıyor. | The detective is wetting the doctor. | Dedektifi doktor ıslatıyor. | The doctor is wetting the detective. |
| 7 | Dedektif astronotu gıdıklıyor. | The detective is tickling the astronaut. | Dedektifi astronot gıdıklıyor. | The astronaut is tickling the detective. |
| 8 | Dedektif futbolcuyu tekmeliyor. | The detective is kicking the football player. | Dedektifi futbolcu tekmeliyor. | The football player is kicking the detective. |
| 9 | Palyaço doktoru gıdıklıyor. | The clown is tickling the doctor. | Palyaçoyu doktor gıdıklıyor. | The doctor is tickling the clown. |
| 10 | Palyaço astronotu tekmeliyor. | The clown is kicking the astronaut. | Palyaçoyu astronot tekmeliyor. | The astronaut is kicking the clown. |
| 11 | Palyaço futbolcuyu çekiyor. | The clown is pulling the football player. | Palyaçoyu futbolcu çekiyor. | The football player is pulling the clown. |
| 12 | Palyaço askeri ısırıyor. | The clown is biting the soldier. | Palyaçoyu asker ısırıyor. | The soldier is biting the clown. |
| 13 | Doktor astronotu çekiyor. | The doctor is pulling the astronaut. | Doktoru astronot çekiyor. | The astronaut is pulling the doctor. |
| 14 | Doktor futbolcuyu ısırıyor. | The doctor is biting the football player. | Doktoru futbolcu ısırıyor. | The football player is biting the doctor. |
| 15 | Doktor askeri itiyor. | The doctor is pushing the soldier. | Doktoru asker itiyor. | The soldier is pushing the doctor. |
| 16 | Doktor hırsızı öpüyor. | The doctor is kissing the thief. | Doktoru hırsız öpüyor. | The thief is kissing the doctor. |
| 17 | Astronot futbolcuyu itiyor. | The astronaut is pushing the football player. | Astronotu futbolcu itiyor. | The football player is pushing the astronaut. |
| 18 | Astronot askeri öpüyor. | The astronaut is kissing the soldier. | Astronotu asker öpüyor. | The soldier is kissing the astronaut. |
| 19 | Astronot hırsızı tutuyor. | The astronaut is holding the thief. | Astronotu hırsız tutuyor. | The thief is holding the astronaut. |
| 20 | Astronot aşçıyı yıkıyor. | The astronaut is washing the chef. | Astronotu aşçı yıkıyor. | The chef is washing the astronaut. |
| 21 | Futbolcu askeri tutuyor. | The football player is holding the soldier. | Futbolcuyu asker tutuyor. | The soldier is holding the football player. |
| 22 | Futbolcu hırsızı yıkıyor. | The football player is washing the thief. | Futbolcuyu hırsız yıkıyor. | The thief is washing the football player. |
| 23 | Futbolcu aşçıyı bağlıyor. | The football player is tying the chef. | Futbolcuyu aşçı bağlıyor. | The chef is tying the football player. |
| 24 | Futbolcu dalgıcı sürüklüyor. | The football player is dragging the diver. | Futbolcuyu dalgıç sürüklüyor. | The diver is dragging the football player. |
| 25 | Asker hırsızı bağlıyor. | The soldier is tying the thief. | Askeri hırsız bağlıyor. | The thief is tying the soldier. |
| 26 | Asker aşçıyı sürüklüyor. | The soldier is dragging the chef. | Askeri aşçı sürüklüyor. | The chef is dragging the soldier. |
| 27 | Asker dalgıcı boyuyor. | The soldier is painting the diver. | Askeri dalgıç boyuyor. | The diver is painting the soldier. |
| 28 | Asker itfaiyeciyi ıslatıyor. | The soldier is wetting the firefighter. | Askeri itfaiyeci ıslatıyor. | The firefighter is wetting the soldier. |
| 29 | Hırsız aşçıyı boyuyor. | The thief is painting the chef. | Hırsızı aşçı boyuyor. | The chef is painting the thief. |
| 30 | Hırsız dalgıcı ıslatıyor. | The thief is wetting the diver. | Hırsızı dalgıç ıslatıyor. | The diver is wetting the thief. |
| 31 | Hırsız itfaiyeciyi gıdıklıyor. | The thief is tickling the firefighter. | Hırsızı itfaiyeci gıdıklıyor. | The firefighter is tickling the thief. |
| 32 | Hırsız korsanı tekmeliyor. | The thief is kicking the pirate. | Hırsızı korsan tekmeliyor. | The pirate is kicking the thief. |
| 33 | Aşçı dalgıcı gıdıklıyor. | The chef is tickling the diver. | Aşçıyı dalgıç gıdıklıyor. | The diver is tickling the chef. |
| 34 | Aşçı itfaiyeciyi tekmeliyor. | The chef is kicking the firefighter. | Aşçıyı itfaiyeci tekmeliyor. | The firefighter is kicking the chef. |
| 35 | Aşçı korsanı çekiyor. | The chef is pulling the pirate. | Aşçıyı korsan çekiyor. | The pirate is pulling the chef. |
| 36 | Aşçı polisi ısırıyor. | The chef is biting the police officer. | Aşçıyı polis ısırıyor. | The police officer is biting the chef. |
| 37 | Dalgıç itfaiyeciyi çekiyor. | The diver is pulling the firefighter. | Dalgıcı itfaiyeci çekiyor. | The firefighter is pulling the diver. |
| 38 | Dalgıç korsanı ısırıyor. | The diver is biting the pirate. | Dalgıcı korsan ısırıyor. | The pirate is biting the diver. |
| 39 | Dalgıç polisi itiyor. | The diver is pushing the police officer. | Dalgıcı polis itiyor. | The police officer is pushing the diver. |
| 40 | Dalgıç dedektifi öpüyor. | The diver is kissing the detective. | Dalgıcı dedektif öpüyor. | The detective is kissing the diver. |
| 41 | İtfaiyeci korsanı itiyor. | The firefighter is pushing the pirate. | İtfaiyeciyi korsan itiyor. | The pirate is pushing the firefighter. |
| 42 | İtfaiyeci polisi öpüyor. | The firefighter is kissing the police officer. | İtfaiyeciyi polis öpüyor. | The police officer is kissing the firefighter. |
| 43 | İtfaiyeci dedektifi tutuyor. | The firefighter is holding the detective. | İtfaiyeciyi dedektif tutuyor. | The detective is holding the firefighter. |
| 44 | İtfaiyeci palyaçoyu yıkıyor. | The firefighter is washing the clown. | İtfaiyeciyi palyaço yıkıyor. | The clown is washing the firefighter. |
| 45 | Korsan polisi tutuyor. | The pirate is holding the police officer. | Korsanı polis tutuyor. | The police officer is holding the pirate. |
| 46 | Korsan dedektifi yıkıyor. | The pirate is washing the detective. | Korsanı dedektif yıkıyor. | The detective is washing the pirate. |
| 47 | Korsan palyaçoyu bağlıyor. | The pirate is tying the clown. | Korsanı palyaço bağlıyor. | The clown is tying the pirate. |
| 48 | Korsan doktoru sürüklüyor. | The pirate is dragging the doctor. | Korsanı doktor sürüklüyor. | The doctor is dragging the pirate. |

*Note.* Canonical SOV = subject–object–verb order; noncanonical OSV = object–subject–verb order. English translations reflect the thematic-role assignment indicated by Turkish case marking.

**Supplementary Table S2.** Electrode-specific retention of artifact-free epochs in the posterior ROI.

| **Electrode** | **Adults** | | **Children** | |
| --- | --- | --- | --- | --- |
|  | **Canonical SOV**  **M (SD) %** | **Noncanonical OSV**  **M (SD) %** | **Canonical SOV**  **M (SD) %** | **Noncanonical OSV**  **M (SD) %** |
| P3 | 99.6 (1.3) | 99.9 (0.4) | 93.1 (16.4) | 91.1 (19.1) |
| Pz | 99.7 (1.0) | 99.7 (1.6) | 91.2 (19.9) | 88.5 (24.8) |
| P4 | 99.7 (1.2) | 99.8 (1.3) | 94.2 (16.1) | 91.7 (19.1) |
| O1 | 99.3 (2.2) | 99.5 (1.8) | 90.9 (18.8) | 87.0 (24.5) |
| Oz | 99.3 (1.8) | 99.8 (0.9) | 89.8 (20.7) | 86.1 (26.2) |
| O2 | 99.0 (2.3) | 99.5 (1.5) | 89.1 (20.3) | 87.3 (25.9) |
| Across posterior ROI | 99.4 (1.3) | 99.7 (1.0) | 91.4 (17.3) | 88.6 (21.5) |

Note. Values are the mean percentage of available trials retained after artifact rejection, with standard deviations in parentheses. Adults had 48 available trials per sentence type. For children, percentages were calculated relative to the number of trials actually presented: 48 per sentence type for the 30 children who completed both experimental blocks and 24 per sentence type for the 7 children who completed one block. The posterior ROI comprised P3, Pz, P4, O1, Oz, and O2. The “Across posterior ROI” row was calculated by first averaging retention across the six electrodes within each participant and then summarizing these participant-level averages.

**Supplementary Table S3.** Descriptive characteristics of child assessments and parental education.

**Panel A. Continuous child measures**

| **Measure** | ***n*** | ***M*** | ***SD*** | **Minimum** | **Maximum** |
| --- | --- | --- | --- | --- | --- |
| Age (years) | 37 | 6.83 | 1.20 | 4.33 | 9.67 |
| Syntactic understanding (raw score) | 37 | 20.41 | 6.64 | 2 | 30 |
| Sentence imitation (raw score) | 37 | 17.51 | 9.54 | 0 | 34 |
| Morphological completion (raw score) | 37 | 23.59 | 8.69 | 0.00 | 36.00 |
| Forward digit span level | 21 | 4.76 | 1.09 | 3.00 | 7.00 |

**Panel B. Parental educational attainment**

| **Parent** | **Primary** | **Secondary** | **High school** | **University and above** |
| --- | --- | --- | --- | --- |
| Mother | 0 (0.0%) | 9 (24.3%) | 16 (43.2%) | 12 (32.4%) |
| Father | 1 (2.7%) | 2 (5.4%) | 15 (40.5%) | 19 (51.4%) |

*Note.* Panel A reports descriptive statistics for the continuous child measures. The grammar composite used in the inferential analyses was calculated as the mean of the within-sample standardized scores for syntactic understanding, sentence imitation, and morphological completion. The overall-language composite was calculated analogously across all six TODİL core subtests. Forward digit span level was available for 21 of the 37 children. Panel B reports parental education, coded as 1 = primary, 2 = secondary, 3 = high school, and 4 = university and above. Paternal education was used descriptively only.

**Supplementary Table S4.** Behavioral accuracy and canonical–noncanonical accuracy difference by group.

| **Group** | ***n*** | **Canonical SOV accuracy *M* (*SD*) [95% CI]** | **Noncanonical OSV accuracy *M* (*SD*) [95% CI]** | **SOV − OSV accuracy difference *M* (*SD*) [95% CI]** |
| --- | --- | --- | --- | --- |
| Adults | 67 | 96.64 (3.42) [95.81, 97.48] | 93.87 (7.50) [92.04, 95.70] | 2.77 (7.70) [0.89, 4.64] |
| Children | 35 | 76.56 (16.78) [70.80, 82.33] | 67.65 (15.71) [62.25, 73.05] | 8.91 (10.07) [5.45, 12.37] |

*Note.* Accuracy is expressed as a percentage. The SOV − OSV accuracy difference is expressed in percentage points; positive values indicate lower accuracy for noncanonical OSV sentences.

**Supplementary Table S5.** Results of the Group × Sentence Type model and planned contrasts for comprehension accuracy.

**Panel A. Omnibus tests**

| **Effect** | ***df*** | ***F*** | ***p*** |
| --- | --- | --- | --- |
| Group | 1, 100 | 131.26 | < .001 |
| Sentence type | 1, 100 | 42.63 | < .001 |
| Group × Sentence Type | 1, 100 | 11.80 | < .001 |

**Panel B. Planned contrasts**

| **Contrast** | ***b*** | ***SE*** | **95% CI** | ***df*** | ***t*** | ***p*Holm** |
| --- | --- | --- | --- | --- | --- | --- |
| Canonical SOV − Noncanonical OSV, adults | 2.77 | 1.05 | [0.69, 4.85] | 100 | 2.64 | .010 |
| Canonical SOV − Noncanonical OSV, children | 8.91 | 1.45 | [6.04, 11.79] | 100 | 6.15 | < .001 |
| Child SOV−OSV difference − adult SOV−OSV difference | 6.14 | 1.79 | [2.60, 9.69] | 100 | 3.43 | < .001 |
| Adults − children, canonical SOV | 20.08 | 2.21 | [15.71, 24.45] | 137.74 | 9.09 | < .001 |
| Adults − children, noncanonical OSV | 26.22 | 2.21 | [21.85, 30.59] | 137.74 | 11.87 | < .001 |

*Note.* Accuracy estimates and coefficients are expressed in percentage points. Panel A reports omnibus tests from the Group × Sentence Type model. Panel B reports the planned contrasts, including the sentence-type contrast within each group, the group contrast within each sentence type, and the child–adult difference in the SOV − OSV accuracy difference. All tests were two-tailed. Holm correction was applied separately across the two group-specific sentence-type contrasts and across the two condition-specific group contrasts. The child–adult difference in the SOV − OSV accuracy difference constituted a single contrast and therefore required no multiplicity adjustment.

**Supplementary Table S6.** Omnibus tests from the Group × Sentence Type models of posterior ERP amplitude.

| **Time window (ms)** | **Effect** | ***F*(1, 102)** | ***p*** | ***p*FDR** |
| --- | --- | --- | --- | --- |
| 600-700 | Group | 6.591 | .012 | .035 |
|  | Sentence type | 2.377 | .126 | .407 |
|  | Group × Sentence Type | 0.306 | .581 | .887 |
| 700-800 | Group | 4.763 | .031 | .042 |
|  | Sentence type | 1.247 | .267 | .407 |
|  | Group × Sentence Type | 0.373 | .543 | .887 |
| 800-900 | Group | 4.798 | .031 | .042 |
|  | Sentence type | 0.194 | .661 | .661 |
|  | Group × Sentence Type | 4.445 | .037 | .225 |
| 900-1000 | Group | 2.330 | .130 | .130 |
|  | Sentence type | 0.893 | .347 | .416 |
|  | Group × Sentence Type | 0.000 | .989 | .989 |
| 1000-1100 | Group | 4.558 | .035 | .042 |
|  | Sentence type | 1.222 | .272 | .407 |
|  | Group × Sentence Type | 0.151 | .698 | .887 |
| 1100-1200 | Group | 13.658 | < .001 | .002 |
|  | Sentence type | 2.228 | .139 | .407 |
|  | Group × Sentence Type | 0.111 | .739 | .887 |

*Note.* Separate models were fitted for each of the six prespecified 100-ms windows at the posterior ROI (P3, Pz, P4, O1, Oz, and O2). BH-FDR correction was performed separately for each omnibus term across the six windows. All omnibus tests were two-tailed.

**Supplementary Table S7.** Directional noncanonical OSV − canonical SOV ERP contrasts within adults and children.

| **Time window (ms)** | **Group** | ***b*** | ***SE*** | **95% CI** | ***t*(102)** | **Directional *p*** | **Directional *p*FDR** |
| --- | --- | --- | --- | --- | --- | --- | --- |
| 600-700 | Adults | 0.522 | 0.166 | [0.193, 0.852] | 3.14 | .001 | .007 |
|  | Children | 0.246 | 0.470 | [-0.686, 1.179] | 0.52 | .301 | .463 |
| 700-800 | Adults | 0.379 | 0.162 | [0.057, 0.700] | 2.34 | .011 | .021 |
|  | Children | 0.111 | 0.407 | [-0.697, 0.919] | 0.27 | .393 | .471 |
| 800-900 | Adults | 0.379 | 0.182 | [0.017, 0.740] | 2.08 | .020 | .030 |
|  | Children | -0.579 | 0.416 | [-1.404, 0.246] | -1.39 | .916 | .916 |
| 900-1000 | Adults | 0.339 | 0.216 | [-0.090, 0.768] | 1.57 | .060 | .072 |
|  | Children | 0.348 | 0.694 | [-1.028, 1.725] | 0.50 | .308 | .463 |
| 1000-1100 | Adults | 0.252 | 0.218 | [-0.181, 0.685] | 1.15 | .126 | .126 |
|  | Children | 0.525 | 0.668 | [-0.800, 1.849] | 0.79 | .217 | .463 |
| 1100-1200 | Adults | 0.525 | 0.215 | [0.098, 0.952] | 2.44 | .008 | .021 |
|  | Children | 0.333 | 0.533 | [-0.724, 1.391] | 0.63 | .267 | .463 |

*Note.* Positive estimates indicate greater positivity for noncanonical OSV than canonical SOV sentences. Directional tests evaluated the prespecified OSV > SOV hypothesis. BH-FDR correction was conducted separately within adults and children across the six posterior time-window contrasts.

**Supplementary Table S8.** Two-tailed adult–child comparisons of posterior ERP amplitude within each sentence type.

| **Time window (ms)** | **Sentence type** | **Adults − children, *b*** | ***SE*** | **95% CI** | ***t*(102)** | ***p*** | ***p*FDR** |
| --- | --- | --- | --- | --- | --- | --- | --- |
| 600-700 | Canonical SOV | 0.632 | 0.403 | [-0.167, 1.431] | 1.57 | .120 | .205 |
|  | Noncanonical OSV | 0.908 | 0.377 | [0.161, 1.655] | 2.41 | .018 | .053 |
| 700-800 | Canonical SOV | 0.529 | 0.388 | [-0.240, 1.298] | 1.36 | .175 | .231 |
|  | Noncanonical OSV | 0.797 | 0.361 | [0.081, 1.513] | 2.21 | .030 | .059 |
| 800-900 | Canonical SOV | 0.292 | 0.397 | [-0.496, 1.081] | 0.74 | .464 | .464 |
|  | Noncanonical OSV | 1.250 | 0.439 | [0.378, 2.121] | 2.84 | .005 | .021 |
| 900-1000 | Canonical SOV | 0.694 | 0.529 | [-0.355, 1.742] | 1.31 | .193 | .231 |
|  | Noncanonical OSV | 0.684 | 0.626 | [-0.558, 1.926] | 1.09 | .277 | .302 |
| 1000-1100 | Canonical SOV | 1.166 | 0.509 | [0.156, 2.175] | 2.29 | .024 | .058 |
|  | Noncanonical OSV | 0.893 | 0.673 | [-0.442, 2.227] | 1.33 | .187 | .231 |
| 1100-1200 | Canonical SOV | 1.613 | 0.503 | [0.614, 2.611] | 3.20 | .002 | .015 |
|  | Noncanonical OSV | 1.805 | 0.583 | [0.649, 2.960] | 3.10 | .003 | .015 |

*Note.* Positive estimates indicate greater positivity in adults than children. BH-FDR correction was applied across all 12 condition-specific adult–child comparisons (2 sentence types × 6 windows). All tests were two-tailed.

**Supplementary Table S9.** Participant-level sensitivity analysis relating condition-specific behavioral accuracy to children’s posterior ERP amplitude.

**Panel A. Omnibus tests of accuracy and Sentence Type × Accuracy**

| **Time window (ms)** | **Accuracy** | | | **Sentence Type × Accuracy** | | |
| --- | --- | --- | --- | --- | --- | --- |
|  | ***F*(1, 31)** | ***p*** | ***p*FDR** | ***F*(1, 31)** | ***p*** | ***p*FDR** |
| 600-700 | 8.772 | .006 | .031 | 0.580 | .452 | .524 |
| 700-800 | 7.462 | .010 | .031 | 0.415 | .524 | .524 |
| 800-900 | 3.932 | .056 | .113 | 0.898 | .351 | .524 |
| 900-1000 | 0.000 | .986 | .986 | 4.877 | .035 | .208 |
| 1000-1100 | 0.018 | .895 | .986 | 3.497 | .071 | .213 |
| 1100-1200 | 0.132 | .719 | .986 | 2.659 | .113 | .226 |

**Panel B. Condition-specific accuracy slopes and slope differences**

| **Time window (ms)** | **Canonical SOV** | | **Noncanonical OSV** | | **OSV − SOV slope** | |
| --- | --- | --- | --- | --- | --- | --- |
|  | ***b* (*SE*)** | ***p*FDR** | ***b* (*SE*)** | ***p*FDR** | ***b* (*SE*)** | ***p*FDR** |
| 600-700 | 0.629 (0.214) | .042 | 0.412 (0.238) | .224 | -0.217 (0.285) | .524 |
| 700-800 | 0.556 (0.192) | .042 | 0.422 (0.220) | .193 | -0.133 (0.207) | .524 |
| 800-900 | 0.493 (0.190) | .057 | 0.301 (0.254) | .327 | -0.191 (0.202) | .524 |
| 900-1000 | 0.422 (0.296) | .309 | -0.432 (0.389) | .330 | -0.853 (0.386) | .208 |
| 1000-1100 | 0.326 (0.275) | .327 | -0.405 (0.422) | .376 | -0.731 (0.391) | .213 |
| 1100-1200 | 0.354 (0.258) | .309 | -0.162 (0.351) | .648 | -0.516 (0.316) | .226 |

*Note.* *N* = 35. Condition-specific behavioral accuracy was paired with ERP amplitude within sentence type and expressed per 10-percentage-point increase after centering. All models also included Sentence Type × Age. Panel A reports omnibus tests of accuracy and Sentence Type × Accuracy; BH-FDR correction was applied separately to each omnibus term across the six time windows. Panel B reports the condition-specific accuracy slopes and the corresponding Sentence Type × Accuracy slope differences. The 12 condition-specific accuracy slopes were corrected together, whereas the six Sentence Type × Accuracy interactions were corrected separately. This analysis tests whether participant-level condition-specific accuracy is associated with condition-specific ERP amplitude.

**Supplementary Table S10.** Child posterior ERP condition effects in sample-restriction sensitivity analyses.

**Panel A. Children with behavioral data (*n* = 35)**

| **Time window (ms)** | **OSV − SOV estimate** | **SE** | **95% CI** | ***t*(100)** | **Directional *p*** | **Directional *p*FDR** |
| --- | --- | --- | --- | --- | --- | --- |
| 600-700 | 0.164 | 0.486 | [−0.801, 1.129] | 0.337 | .369 | .678 |
| 700-800 | −0.068 | 0.401 | [−0.863, 0.727] | −0.170 | .567 | .681 |
| 800-900 | −0.786 | 0.383 | [−1.545, −0.026] | −2.052 | .979 | .979 |
| 900-1000 | 0.087 | 0.717 | [−1.336, 1.510] | 0.121 | .452 | .678 |
| 1000-1100 | 0.302 | 0.694 | [−1.076, 1.679] | 0.434 | .332 | .678 |
| 1100-1200 | 0.119 | 0.544 | [−0.961, 1.199] | 0.219 | .413 | .678 |

**Panel B. Children completing both experimental blocks (*n* = 30)**

| **Time window (ms)** | **OSV − SOV estimate** | **SE** | **95% CI** | ***t*(95)** | **Directional *p*** | **Directional *p*FDR** |
| --- | --- | --- | --- | --- | --- | --- |
| 600-700 | 0.002 | 0.462 | [−0.916, 0.920] | 0.005 | .498 | .918 |
| 700-800 | 0.025 | 0.485 | [−0.938, 0.989] | 0.052 | .479 | .918 |
| 800-900 | −0.714 | 0.510 | [−1.727, 0.298] | −1.400 | .918 | .918 |
| 900-1000 | −0.468 | 0.616 | [−1.690, 0.754] | −0.760 | .775 | .918 |
| 1000-1100 | −0.421 | 0.550 | [−1.514, 0.671] | −0.766 | .777 | .918 |
| 1100-1200 | −0.366 | 0.560 | [−1.478, 0.747] | −0.653 | .742 | .918 |

*Note.* Panel A reports the posterior noncanonical OSV − canonical SOV condition effect in children with available behavioral data (*n* = 35), and Panel B reports the corresponding effect in children who completed both experimental blocks (*n* = 30). Estimates are mean-amplitude differences in µV; positive values indicate greater positivity for noncanonical OSV than canonical SOV sentences. Directional *p* values test the prespecified hypothesis of greater positivity for noncanonical OSV than canonical SOV sentences. BH-FDR correction was applied across the six time windows separately within each restricted sample. Confidence intervals are two-sided 95% intervals.

**Supplementary Table S11.** Age-related associations with comprehension accuracy in children.

**Panel A. Omnibus tests from the linear age model**

| **Effect** | ***df*** | ***F*** | ***p*** |
| --- | --- | --- | --- |
| Sentence type | 1, 33 | 26.68 | < .001 |
| Age | 1, 33 | 1.36 | .252 |
| Sentence Type × Age | 1, 33 | 0.08 | .777 |

**Panel B. Condition-specific linear age slopes**

| **Effect** | ***b*** | ***SE*** | **95% CI** | ***df*** | ***t*** | ***p*** |
| --- | --- | --- | --- | --- | --- | --- |
| Age slope, canonical SOV | 2.72 | 2.27 | [-1.88, 7.31] | 40.19 | 1.20 | .239 |
| Age slope, noncanonical OSV | 2.31 | 2.27 | [-2.28, 6.91] | 40.19 | 1.02 | .316 |
| Noncanonical OSV − canonical SOV age slope | -0.41 | 1.43 | [-3.32, 2.51] | 33 | -0.29 | .777 |

**Panel C. Omnibus tests from the quadratic age sensitivity model**

| **Effect** | ***df*** | ***F*** | ***p*** |
| --- | --- | --- | --- |
| Age² | 1, 32 | 0.83 | .370 |
| Sentence Type × Age² | 1, 32 | 0.40 | .529 |

**Panel D. Descriptive behavioral accuracy by age interval**

| **Age interval** | ***n*** | **Canonical SOV *M* (*SD*)** | **Noncanonical OSV *M* (*SD*)** | **SOV − OSV difference *M* (*SD*)** |
| --- | --- | --- | --- | --- |
| 4-5 years | 10 | 73.56 (19.30) | 65.33 (14.01) | 8.23 (9.40) |
| 6-7 years | 18 | 76.76 (17.84) | 67.13 (18.16) | 9.63 (11.95) |
| 8-9 years | 7 | 80.36 (10.12) | 72.32 (11.65) | 8.04 (5.82) |

*Note.* Age was centered in years for the inferential models. Panel A reports omnibus tests from the linear age model. In Panel B, coefficients represent percentage-point changes in accuracy per one-year increase in age. The quadratic sensitivity model retained both linear and quadratic age terms and their interactions with sentence type; Panel C displays only the additional quadratic terms. Panel D presents descriptive results by age interval; these intervals were not used for subgroup inferential tests.

**Supplementary Figure S1.** Individual behavioral accuracy and posterior ERP values across child age.

*Note.* Panel A shows individual condition-specific comprehension accuracy values with descriptive quadratic fits for canonical SOV and noncanonical OSV sentences. Panel B shows individual posterior noncanonical OSV − canonical SOV amplitude differences for each of the six prespecified 100-ms windows, with descriptive quadratic fits. The fitted curves are visual summaries and are not additional inferential models.

**Supplementary Table S12.** Linear age associations with children’s posterior ERP amplitude.

**Panel A. Omnibus tests of Age and Sentence Type × Age**

| **Time window (ms)** | **Age** | | | **Sentence Type × Age** | | |
| --- | --- | --- | --- | --- | --- | --- |
|  | ***F*(1, 35)** | ***p*** | ***p*FDR** | ***F*(1, 35)** | ***p*** | ***p*FDR** |
| 600-700 | 0.61 | .441 | .441 | 0.04 | .843 | .948 |
| 700-800 | 1.96 | .171 | .205 | 1.31 | .260 | .828 |
| 800-900 | 3.18 | .083 | .152 | 0.36 | .552 | .828 |
| 900-1000 | 2.83 | .101 | .152 | 0.00 | .948 | .948 |
| 1000-1100 | 3.30 | .078 | .152 | 0.52 | .476 | .828 |
| 1100-1200 | 4.84 | .034 | .152 | 0.89 | .352 | .828 |

**Panel B. Condition-specific age slopes and slope differences**

| **Time window (ms)** | **Canonical SOV** | | **Noncanonical OSV** | | **OSV − SOV slope** | |
| --- | --- | --- | --- | --- | --- | --- |
|  | ***b* (*SE*)** | ***p*FDR** | ***b* (*SE*)** | ***p*FDR** | ***b* (*SE*)** | ***p*FDR** |
| 600-700 | 0.164 (0.320) | .611 | 0.237 (0.309) | .527 | 0.072 (0.362) | .948 |
| 700-800 | 0.544 (0.305) | .217 | 0.220 (0.310) | .527 | -0.324 (0.283) | .828 |
| 800-900 | 0.496 (0.317) | .217 | 0.669 (0.393) | .217 | 0.173 (0.289) | .828 |
| 900-1000 | 0.674 (0.399) | .217 | 0.640 (0.528) | .312 | -0.034 (0.518) | .948 |
| 1000-1100 | 0.563 (0.380) | .221 | 0.930 (0.568) | .217 | 0.367 (0.509) | .828 |
| 1100-1200 | 0.639 (0.371) | .217 | 1.028 (0.484) | .217 | 0.389 (0.412) | .828 |

*Note.* *N* = 37. Age was centered in years for the inferential models. Panel A reports omnibus tests of Age and Sentence Type × Age; BH-FDR correction was applied separately to each omnibus term across the six time windows. Panel B reports the condition-specific age slopes and the corresponding Sentence Type × Age slope differences. Coefficients represent changes in posterior ERP amplitude, in µV, associated with a one-year increase in age. The 12 condition-specific age slopes were corrected together, whereas the six Sentence Type × Age interactions were corrected separately. All tests were two-tailed.

**Supplementary Table S13.** Quadratic age sensitivity and age-interval descriptives for children’s posterior ERP response.

**Panel A. Omnibus tests of Age² and Sentence Type × Age²**

| **Time window (ms)** | **Age² *F*(1, 34)** | ***p*** | ***p*FDR** | **Sentence Type × Age² *F*(1, 34)** | ***p*** | ***p*FDR** |
| --- | --- | --- | --- | --- | --- | --- |
| 600-700 | 0.56 | .459 | .4594 | 0.00 | .963 | .9629 |
| 700-800 | 4.82 | .035 | .0882 | 0.01 | .946 | .9629 |
| 800-900 | 7.85 | .008 | .0501 | 0.82 | .372 | .9629 |
| 900-1000 | 4.37 | .044 | .0882 | 0.01 | .925 | .9629 |
| 1000-1100 | 1.79 | .190 | .2281 | 0.03 | .865 | .9629 |
| 1100-1200 | 3.09 | .088 | .1316 | 0.25 | .621 | .9629 |

**Panel B. Descriptive noncanonical OSV − canonical SOV amplitudes by age interval**

| **Age interval** | ***n*** | **600–700 ms *M* (*SD*)** | **700–800 ms *M* (*SD*)** | **800–900 ms *M* (*SD*)** | **900–1000 ms *M* (*SD*)** | **1000–1100 ms *M* (*SD*)** | **1100–1200 ms *M* (*SD*)** |
| --- | --- | --- | --- | --- | --- | --- | --- |
| 4-5 years | 11 | 0.410 (1.752) | 0.775 (1.673) | -0.633 (2.042) | 0.200 (3.080) | -0.009 (3.375) | -0.088 (2.898) |
| 6-7 years | 19 | 0.245 (3.175) | 0.130 (2.364) | -0.409 (2.197) | 0.819 (4.344) | 1.015 (3.864) | 0.813 (3.118) |
| 8-9 years | 7 | -0.008 (2.051) | -0.985 (1.248) | -0.952 (1.960) | -0.695 (2.631) | 0.033 (3.793) | -0.306 (2.828) |

*Note.* *N* = 37 for the quadratic age models. Panel A reports the additional Age² and Sentence Type × Age² terms from models that also included linear age and its interaction with sentence type. BH-FDR correction was applied separately to each model term across the six time windows. Four decimal places are shown for adjusted *p* values in Panel A because the Age² effect at 800–900 ms lies immediately above .05 (*p*FDR = .0501). Panel B reports descriptive noncanonical OSV − canonical SOV mean-amplitude differences, in µV, by age interval across the six prespecified time windows; positive values indicate greater positivity for noncanonical OSV than canonical SOV sentences. The age intervals were not used for subgroup inferential tests.

**Supplementary Table S14.** Grammar and overall language as predictors of comprehension accuracy in children beyond age.

**Panel A. Omnibus tests from the grammar model**

| **Effect** | ***df*** | ***F*** | ***p*** |
| --- | --- | --- | --- |
| Sentence type | 1, 32 | 27.46 | < .001 |
| Age | 1, 32 | 3.67 | .064 |
| Grammar | 1, 32 | 42.16 | < .001 |
| Sentence Type × Age | 1, 32 | 0.24 | .629 |
| Sentence Type × Grammar | 1, 32 | 1.97 | .171 |

**Panel B. Condition-specific age and grammar slopes and slope differences**

| **Predictor and effect** | ***b*** | ***SE*** | **95% CI** | ***df*** | ***t*** | ***p*** |
| --- | --- | --- | --- | --- | --- | --- |
| Age slope, canonical SOV | -3.64 | 1.88 | [-7.43, 0.15] | 46.56 | -1.93 | .059 |
| Age slope, noncanonical OSV | -2.83 | 1.88 | [-6.62, 0.96] | 46.56 | -1.50 | .139 |
| Noncanonical OSV − canonical SOV age slope | 0.81 | 1.66 | [-2.57, 4.19] | 32 | 0.49 | .629 |
| Grammar slope, canonical SOV | 16.20 | 2.51 | [11.15, 21.26] | 46.56 | 6.45 | < .001 |
| Grammar slope, noncanonical OSV | 13.10 | 2.51 | [8.04, 18.16] | 46.56 | 5.21 | < .001 |
| Noncanonical OSV − canonical SOV grammar slope | -3.10 | 2.21 | [-7.61, 1.41] | 32 | -1.40 | .171 |

**Panel C. Omnibus tests from the overall-language robustness model**

| **Effect** | ***df*** | ***F*** | ***p*** |
| --- | --- | --- | --- |
| Sentence type | 1, 32 | 26.95 | < .001 |
| Age | 1, 32 | 3.28 | .079 |
| Overall language | 1, 32 | 33.67 | < .001 |
| Sentence Type × Age | 1, 32 | 0.16 | .695 |
| Sentence Type × Overall Language | 1, 32 | 1.34 | .256 |

**Panel D. Condition-specific age and overall-language slopes and slope differences**

| **Predictor and effect** | ***b*** | ***SE*** | **95% CI** | ***df*** | ***t*** | ***p*** |
| --- | --- | --- | --- | --- | --- | --- |
| Age slope, canonical SOV | -3.65 | 2.02 | [-7.72, 0.41] | 45.27 | -1.81 | .077 |
| Age slope, noncanonical OSV | -2.98 | 2.02 | [-7.04, 1.09] | 45.27 | -1.48 | .147 |
| Noncanonical OSV − canonical SOV age slope | 0.68 | 1.71 | [-2.80, 4.15] | 32 | 0.40 | .695 |
| Overall-language slope, canonical SOV | 15.85 | 2.76 | [10.30, 21.40] | 45.27 | 5.75 | < .001 |
| Overall-language slope, noncanonical OSV | 13.15 | 2.76 | [7.60, 18.70] | 45.27 | 4.77 | < .001 |
| Noncanonical OSV − canonical SOV overall-language slope | -2.70 | 2.33 | [-7.44, 2.05] | 32 | -1.16 | .256 |

*Note.* *N* = 35. Accuracy and coefficients are expressed in percentage points. Panels A and B report the model including Age, Grammar, and their respective interactions with Sentence Type. Panels C and D report the analogous robustness model in which the grammar composite was replaced by the overall-language composite. Age was centered in years; age coefficients in Panels B and D represent percentage-point changes in accuracy per one-year increase in age. The grammar and overall-language composites were constructed from within-sample standardized subtest scores; their coefficients represent percentage-point changes in accuracy per one-unit increase in the respective composite. Panels A and C report omnibus tests, whereas Panels B and D report condition-specific slopes and the corresponding slope differences. All tests were two-tailed.

**Supplementary Table S15.** Forward digit span sensitivity analyses for children’s comprehension accuracy.

**Panel A. Omnibus tests from the forward-digit-span model**

| **Effect** | ***df*** | ***F*** | ***p*** |
| --- | --- | --- | --- |
| Sentence type | 1, 17 | 15.30 | .001 |
| Age | 1, 17 | 0.01 | .916 |
| Forward digit span | 1, 17 | 13.57 | .002 |
| Sentence Type × Age | 1, 17 | 0.79 | .387 |
| Sentence Type × Forward Digit Span | 1, 17 | 0.06 | .813 |

**Panel B. Condition-specific forward-digit-span slopes and slope difference**

| **Effect** | ***b*** | ***SE*** | **95% CI** | ***df*** | ***t*** | ***p*** |
| --- | --- | --- | --- | --- | --- | --- |
| FDS slope, canonical SOV | 11.94 | 3.35 | [4.98, 18.90] | 20.79 | 3.57 | .002 |
| FDS slope, noncanonical OSV | 11.43 | 3.35 | [4.47, 18.39] | 20.79 | 3.42 | .003 |
| OSV − SOV FDS slope | -0.51 | 2.13 | [-5.01, 3.99] | 17 | -0.24 | .813 |

**Panel C. Grammar robustness in the forward-digit-span-complete sample**

| **Model** | **Grammar** | | **FDS** | | **Sentence Type × Grammar** | |
| --- | --- | --- | --- | --- | --- | --- |
|  | ***F*** | ***p*** | ***F*** | ***p*** | ***F*** | ***p*** |
| Grammar model on FDS-complete sample | 38.44 | < .001 | — | — | 0.30 | .588 |
| Grammar model adjusted for FDS | 15.13 | .001 | 1.17 | .296 | 0.30 | .588 |

**Panel D. Condition-specific grammar slopes before and after forward-digit-span adjustment**

| **Model** | **Canonical SOV** | | **Noncanonical OSV** | | **OSV − SOV** | |
| --- | --- | --- | --- | --- | --- | --- |
|  | **Grammar slope *b* (*SE*)** | ***p*** | **Grammar slope *b* (*SE*)** | ***p*** | **Grammar slope *b* (*SE*)** | ***p*** |
| Baseline | 17.62 (3.00) | < .001 | 16.26 (3.00) | < .001 | -1.36 (2.46) | .588 |
| Adjusted for FDS | 14.96 (3.87) | < .001 | 13.60 (3.87) | .002 | -1.36 (2.46) | .588 |

*Note.* Forward digit span (FDS) was standardized within the children with available data and is interpreted as a measure of verbal short-term memory. Panels A and B report analyses of the 20 children with both FDS and behavioral data. Panel A reports omnibus tests from the model including Age, FDS, and their respective interactions with Sentence Type, whereas Panel B reports the condition-specific FDS slopes and their difference. Panels C and D evaluate whether the association between grammar and comprehension remains after adjustment for FDS using the same FDS-complete sample (*n* = 20), thereby holding sample composition constant. Panel C compares the omnibus Grammar and Sentence Type × Grammar effects before and after FDS adjustment and reports the FDS main effect in the adjusted model. Panel D reports the corresponding condition-specific grammar slopes and slope differences. All tests were two-tailed.

**Supplementary Table S16.** Maternal education, grammatical ability, and comprehension accuracy in children.

**Panel A. Maternal education predicting the grammar composite beyond age**

| **Predictor** | ***b*** | ***SE*** | ***T*** | ***p*** |
| --- | --- | --- | --- | --- |
| Age | 0.349 | 0.098 | 3.58 | .001 |
| Maternal education | 0.580 | 0.155 | 3.75 | < .001 |

**Panel B. Omnibus tests of grammar and maternal education predicting comprehension accuracy**

| **Effect** | ***df*** | ***F*** | ***p*** |
| --- | --- | --- | --- |
| Sentence type | 1, 32 | 27.46 | < .001 |
| Age | 1, 31 | 3.46 | .073 |
| Grammar | 1, 31 | 30.97 | < .001 |
| Maternal education | 1, 31 | 0.00 | .992 |
| Sentence Type × Age | 1, 32 | 0.24 | .629 |
| Sentence Type × Grammar | 1, 32 | 1.97 | .171 |

**Panel C. Condition-specific grammar slopes after maternal-education adjustment**

| **Effect** | ***b*** | ***SE*** | **95% CI** | ***df*** | ***t*** | ***p*** |
| --- | --- | --- | --- | --- | --- | --- |
| Grammar slope, canonical SOV | 16.19 | 2.85 | [10.43, 21.95] | 41.68 | 5.67 | < .001 |
| Grammar slope, noncanonical OSV | 13.09 | 2.85 | [7.33, 18.85] | 41.68 | 4.59 | < .001 |
| Grammar slope difference, noncanonical OSV − canonical SOV | -3.10 | 2.21 | [-7.61, 1.41] | 32 | -1.40 | .171 |

*Note.* Maternal education was coded from 1 (primary) to 4 (university and above) and mean-centered for inferential analyses. Panel A reports the associations of age and maternal education with the grammar composite in all 37 children. Panel B reports omnibus tests from the comprehension model including Age, Grammar, Maternal Education, Sentence Type × Age, and Sentence Type × Grammar for the 35 children with behavioral data. Panel C reports the condition-specific grammar slopes and their difference from the same maternal-education-adjusted comprehension model. Paternal education was not entered into inferential models. These analyses evaluate whether the grammar–comprehension association is robust to adjustment for maternal educational background.

**Supplementary Table S17.** Exploratory analyses using the TODİL morphological-completion subtest.

**Panel A. Omnibus tests from the morphological-completion behavioral model**

| **Effect** | ***df*** | ***F*** | ***p*** |
| --- | --- | --- | --- |
| Sentence type | 1, 32 | 27.10 | < .001 |
| Age | 1, 32 | 1.70 | .202 |
| Morphological completion | 1, 32 | 27.31 | < .001 |
| Sentence Type × Age | 1, 32 | 0.14 | .708 |
| Sentence Type × Morphological Completion | 1, 32 | 1.52 | .226 |

**Panel B. Condition-specific morphological-completion slopes for accuracy**

| **Effect** | ***b*** | ***SE*** | **95% CI** | ***df*** | ***t*** | ***p*** |
| --- | --- | --- | --- | --- | --- | --- |
| Morphological-completion slope, SOV | 13.31 | 2.52 | [8.23, 18.39] | 44.02 | 5.28 | < .001 |
| Morphological-completion slope, OSV | 10.80 | 2.52 | [5.72, 15.88] | 44.02 | 4.28 | < .001 |
| OSV − SOV morphological-completion slope | -2.51 | 2.04 | [-6.66, 1.64] | 32 | -1.23 | .226 |

**Panel C. Omnibus morphological-completion effects on posterior ERP amplitude**

| **Time window (ms)** | **Morphological completion** | | | **Sentence Type × Morphological completion** | | |
| --- | --- | --- | --- | --- | --- | --- |
|  | ***F*(1, 34)** | ***p*** | ***p*FDR** | ***F*(1, 34)** | ***p*** | ***p*FDR** |
| 600-700 | 1.87 | .181 | .711 | 1.36 | .251 | .352 |
| 700-800 | 1.41 | .244 | .711 | 1.14 | .293 | .352 |
| 800-900 | 0.40 | .532 | .711 | 2.31 | .137 | .352 |
| 900-1000 | 0.33 | .572 | .711 | 4.15 | .050 | .297 |
| 1000-1100 | 0.21 | .652 | .711 | 1.29 | .264 | .352 |
| 1100-1200 | 0.14 | .711 | .711 | 0.47 | .499 | .499 |

**Panel D. Condition-specific morphological-completion ERP slopes and slope differences**

| **Time window (ms)** | **Canonical SOV** | | **Noncanonical OSV** | | **OSV − SOV slope** | |
| --- | --- | --- | --- | --- | --- | --- |
|  | ***b* (*SE*)** | ***p*FDR** | ***b* (*SE*)** | ***p*FDR** | ***b* (*SE*)** | ***p*FDR** |
| 600-700 | 0.764 (0.425) | .554 | 0.187 (0.428) | .886 | -0.577 (0.494) | .352 |
| 700-800 | 0.649 (0.409) | .554 | 0.235 (0.430) | .883 | -0.414 (0.388) | .352 |
| 800-900 | 0.581 (0.429) | .554 | -0.011 (0.547) | .997 | -0.592 (0.389) | .352 |
| 900-1000 | 0.385 (0.552) | .883 | -1.001 (0.715) | .554 | -1.386 (0.681) | .297 |
| 1000-1100 | 0.136 (0.528) | .958 | -0.654 (0.783) | .883 | -0.790 (0.696) | .352 |
| 1100-1200 | -0.002 (0.516) | .997 | -0.391 (0.669) | .883 | -0.389 (0.569) | .499 |

*Note.* Morphological completion was standardized within the child sample and entered as an alternative predictor to the grammar composite, not simultaneously with it. Panels A and B report the behavioral analyses (*n* = 35). Panel A reports omnibus tests from the model including Age, Morphological Completion, and their respective interactions with Sentence Type, whereas Panel B reports the condition-specific morphological-completion slopes and the corresponding OSV − SOV slope difference. Panels C and D report the posterior ERP analyses (*n* = 37). Panel C reports omnibus Morphological Completion and Sentence Type × Morphological Completion effects across the six time windows, and Panel D reports the corresponding condition-specific ERP slopes and slope differences. For the ERP analyses, BH-FDR correction was applied separately across the six morphological-completion main effects, the six Sentence Type × Morphological Completion interactions, and the 12 condition-specific slopes. All tests were two-tailed.

**Supplementary Table S18.** Grammar as a predictor of children’s posterior ERP amplitude beyond age.

**Panel A. Omnibus grammar and Sentence Type × Grammar effects**

| **Time window (ms)** | **Grammar** | | | **Sentence Type × Grammar** | | |
| --- | --- | --- | --- | --- | --- | --- |
|  | ***F*(1, 34)** | ***p*** | ***p*FDR** | ***F*(1, 34)** | ***p*** | ***p*FDR** |
| 600-700 | 1.81 | .187 | .591 | 2.15 | .152 | .156 |
| 700-800 | 1.09 | .303 | .591 | 2.11 | .156 | .156 |
| 800-900 | 0.18 | .676 | .676 | 3.39 | .074 | .137 |
| 900-1000 | 0.77 | .387 | .591 | 6.65 | .014 | .086 |
| 1000-1100 | 0.54 | .469 | .591 | 3.25 | .080 | .137 |
| 1100-1200 | 0.48 | .492 | .591 | 3.01 | .092 | .137 |

**Panel B. Condition-specific grammar slopes and slope differences**

| **Time window (ms)** | **Canonical SOV** | | **Noncanonical OSV** | | **OSV − SOV slope** | |
| --- | --- | --- | --- | --- | --- | --- |
|  | ***b* (*SE*)** | ***p*FDR** | ***b* (*SE*)** | ***p*FDR** | ***b* (*SE*)** | ***p*FDR** |
| 600-700 | 0.896 (0.457) | .404 | 0.121 (0.465) | .827 | -0.775 (0.529) | .156 |
| 700-800 | 0.724 (0.442) | .430 | 0.122 (0.467) | .827 | -0.601 (0.414) | .156 |
| 800-900 | 0.589 (0.466) | .430 | -0.175 (0.592) | .827 | -0.764 (0.415) | .137 |
| 900-1000 | 0.411 (0.597) | .827 | -1.431 (0.757) | .404 | -1.842 (0.714) | .086 |
| 1000-1100 | 0.211 (0.571) | .827 | -1.111 (0.835) | .430 | -1.323 (0.733) | .137 |
| 1100-1200 | 0.123 (0.559) | .827 | -0.910 (0.712) | .430 | -1.032 (0.595) | .137 |

*Note.* *N* = 37. Panel A reports omnibus Grammar and Sentence Type × Grammar effects across the six time windows. Panel B reports the corresponding condition-specific grammar slopes and OSV − SOV slope differences. Coefficients are expressed in µV per one-unit increase in the grammar composite. All models also included Age, Sentence Type × Age, and Sentence Type. BH-FDR correction was applied separately across the six grammar main effects, the six Sentence Type × Grammar interactions, and the 12 condition-specific grammar slopes. All tests were two-tailed.

**Supplementary Table S19.** Overall language as a predictor of children’s posterior ERP amplitude beyond age: robustness analysis.

**Panel A. Omnibus overall-language and Sentence Type × Overall Language effects**

| **Time window (ms)** | **Overall language** | | | **Sentence Type × Overall Language** | | |
| --- | --- | --- | --- | --- | --- | --- |
|  | ***F*(1, 34)** | ***p*** | ***p*FDR** | ***F*(1, 34)** | ***p*** | ***p*FDR** |
| 600-700 | 1.66 | .207 | .608 | 1.30 | .261 | .314 |
| 700-800 | 1.18 | .285 | .608 | 1.04 | .314 | .314 |
| 800-900 | 0.38 | .542 | .608 | 2.40 | .130 | .272 |
| 900-1000 | 0.34 | .564 | .608 | 5.12 | .030 | .181 |
| 1000-1100 | 0.28 | .598 | .608 | 1.94 | .173 | .272 |
| 1100-1200 | 0.27 | .608 | .608 | 1.86 | .181 | .272 |

**Panel B. Condition-specific overall-language slopes and slope differences**

| **Time window (ms)** | **Canonical SOV** | | **Noncanonical OSV** | | **OSV − SOV slope** | |
| --- | --- | --- | --- | --- | --- | --- |
|  | ***b* (*SE*)** | ***p*FDR** | ***b* (*SE*)** | ***p*FDR** | ***b* (*SE*)** | ***p*FDR** |
| 600-700 | 0.832 (0.485) | .559 | 0.190 (0.487) | .886 | -0.641 (0.562) | .314 |
| 700-800 | 0.686 (0.468) | .559 | 0.235 (0.489) | .886 | -0.451 (0.441) | .314 |
| 800-900 | 0.659 (0.488) | .559 | -0.026 (0.622) | .967 | -0.685 (0.442) | .272 |
| 900-1000 | 0.507 (0.626) | .727 | -1.223 (0.809) | .559 | -1.729 (0.764) | .181 |
| 1000-1100 | 0.202 (0.599) | .886 | -0.890 (0.886) | .664 | -1.092 (0.784) | .272 |
| 1100-1200 | 0.124 (0.587) | .909 | -0.742 (0.754) | .664 | -0.866 (0.634) | .272 |

*Note.* *N* = 37. Panel A reports omnibus Overall Language and Sentence Type × Overall Language effects across the six time windows. Panel B reports the corresponding condition-specific overall-language slopes and OSV − SOV slope differences. Coefficients are expressed in µV per one-unit increase in the overall-language composite. The overall-language composite was entered in place of the grammar composite as a robustness analysis. All models also included Age, Sentence Type × Age, and Sentence Type. BH-FDR correction was applied separately across the six overall-language main effects, the six Sentence Type × Overall Language interactions, and the 12 condition-specific overall-language slopes. All tests were two-tailed.

**Additional Lexical Characterization and Age-Restricted Sensitivity Analyses**

**Stimulus-selection criteria.** Developmental appropriateness and visual identifiability were explicit criteria during stimulus preparation. The research team, comprising one PhD candidate and one master’s student in Speech and Language Therapy and one researcher with a PhD in cognitive neuroscience, evaluated the lexical items and corresponding pictures for expected familiarity from approximately age 4 onward and ease of visual representation and identification. Opinions were also obtained from three speech-language therapists with PhDs and professional experience in speech and language intervention with children.

**Lexical-frequency searches.** Item-level lexical frequencies were obtained from the Turkish National Corpus (TNC; Aksan et al., 2012) using the “Biçimbirim arama (başsözcük)” (Morpheme search (headword)) search option. For each lexical item, the relevant stem was entered and the appropriate word category was selected (noun or verb), so that frequencies reflected occurrences associated with the corresponding headword rather than a single surface form (e.g., *doktor* “doctor” was searched as the stem *doktor* with the noun category selected, and *çekmek* “to pull” was searched as the stem *çek* with the verb category selected). Each item was searched first in the full corpus and then with the corpus restricted to spoken texts, and normalized frequency per million words was recorded. The full TNC contains 49,664,176 written and 1,014,023 spoken words. Because the TNC is a general-purpose corpus rather than a child-directed or age-stratified corpus, these frequencies were treated as descriptive indices of lexical occurrence in Turkish rather than direct measures of familiarity or age of acquisition in young children. The verb stem *tekmele* was not attested in the spoken subcorpus.

**Retrospective age-of-acquisition norming.** To obtain an independent developmental estimate of lexical accessibility, retrospective age-of-acquisition (AoA) ratings were collected for the 24 experimental lexical items using a procedure adapted from Kuperman et al. (2012). Respondents were instructed to estimate the age at which they would have understood each word if it had been used in front of them, regardless of whether they themselves could yet use, read, or write it. Ten calibration words spanning the AoA range were presented before the main item block. The main block contained the 24 experimental items together with 24 control words drawn from the Turkish picture-naming database of Eskioğlu et al. (2025). Response options ranged from “1 year or earlier” through individual ages 2–18 and “19 years or later,” with an additional “I do not know” option. “I do not know” responses were treated as missing for item summaries.

The survey yielded 142 responses. Language-background eligibility was evaluated jointly from the reported native/early-acquired language(s), whether Turkish had been acquired from birth or early childhood, and the country in which most of ages 0–7 had been spent. Four respondents whose predominant early-childhood linguistic environment was a country other than Turkey. Multilingual respondents were retained when Turkish was an early-acquired language and the early-childhood environment was primarily in Türkiye. One additional respondent was excluded because their ratings showed a negative rank-order association with the independently normed control words from Eskioğlu et al. (2025) (Spearman ρ = −.11), indicating anomalous response behavior; no fixed correlation threshold was otherwise imposed. The final norming sample therefore comprised 137 respondents (125 women, 12 men; age *M* = 19.51 years, *SD* = 1.74, range = 18–35). As a validity check, mean ratings for the 24 control words correlated strongly with the independent subjective Turkish AoA norms reported by Eskioğlu et al. (2025), Spearman ρ = .981, *p* < .001; the corresponding correlation for the 10 calibration words was ρ = .988, *p* < .001. Item-level AoA estimates for the experimental words are reported in Supplementary Table S20.

**Supplementary Table S20.** Item-level lexical characteristics and retrospective age-of-acquisition ratings for the experimental nouns and verbs.

| **Lexical item** | **Full TNC frequency (per million)** | | **Spoken TNC frequency (per million)** | | **AoA *n*** | | **AoA *M* (*SD*) (years)** | | **AoA median (years)** | |  |
| --- | --- | --- | --- | --- | --- | --- | --- | --- | --- | --- | --- |
| **Nouns** | | | | | | | | | | |  |
| polis | 277.67 | | 209.07 | | 137 | | 3.93 (1.54) | | 4 | |  |
| dedektif | 9.33 | | 2.96 | | 136 | | 6.80 (2.04) | | 7 | |  |
| palyaço | 9.33 | | 0.99 | | 136 | | 4.32 (1.55) | | 4 | |  |
| doktor | 223.96 | | 411.23 | | 137 | | 3.47 (1.49) | | 3 | |  |
| astronot | 4.14 | | 3.94 | | 136 | | 5.90 (2.12) | | 6 | |  |
| futbolcu | 86.70 | | 27.61 | | 137 | | 5.00 (1.95) | | 5 | |  |
| asker | 499.70 | | 356.99 | | 137 | | 4.95 (1.93) | | 5 | |  |
| hırsız | 31.77 | | 34.52 | | 136 | | 5.15 (1.84) | | 5 | |  |
| aşçı | 13.40 | | 9.86 | | 137 | | 5.34 (2.14) | | 5 | |  |
| dalgıç | 4.06 | | 0.99 | | 136 | | 6.74 (2.26) | | 6 | |  |
| itfaiyeci | 1.85 | | 0.99 | | 136 | | 4.87 (2.14) | | 4 | |  |
| korsan | 15.53 | | 7.89 | | 137 | | 5.69 (1.91) | | 5 | |  |
| **Verbs** | | | | | | | | | | | |
| çekmek | | 1046.35 | | 947.71 | | 136 | | 3.61 (1.47) | | 4 | |
| ısırmak | | 23.74 | | 24.65 | | 137 | | 2.97 (1.37) | | 3 | |
| itmek | | 90.91 | | 50.29 | | 137 | | 3.34 (1.41) | | 3 | |
| öpmek | | 126.80 | | 95.66 | | 137 | | 2.58 (1.20) | | 2 | |
| tutmak | | 998.06 | | 687.36 | | 137 | | 3.10 (1.51) | | 3 | |
| yıkamak | | 100.46 | | 97.63 | | 137 | | 3.14 (1.31) | | 3 | |
| bağlamak | | 306.64 | | 162.72 | | 136 | | 4.51 (1.48) | | 5 | |
| sürüklemek | | 75.40 | | 24.65 | | 137 | | 4.65 (1.78) | | 4 | |
| boyamak | | 49.69 | | 72.98 | | 137 | | 3.05 (1.17) | | 3 | |
| ıslatmak | | 39.92 | | 16.76 | | 137 | | 3.85 (1.65) | | 3 | |
| gıdıklamak | | 4.34 | | 5.92 | | 137 | | 3.27 (1.43) | | 3 | |
| tekmelemek | | 4.81 | | Not attested | | 136 | | 4.04 (1.77) | | 4 | |

*Note.* TNC = Turkish National Corpus; AoA = age of acquisition. TNC frequencies are lemma frequencies expressed per million words. For verbs, the AoA item is shown in infinitive form, whereas TNC searches were based on the bare verb stem. The spoken TNC value for *tekmelemek* is reported as not attested because the verb stem *tekmele* was not found. AoA *n* is the number of valid ratings after participant exclusions and omission of “I do not know” responses. The highest median AoA among the 24 experimental items was 7 years (*dedektif*).

**Age-restricted sensitivity analyses.** Because age 7 was at or above the median retrospective AoA of every experimental lexical item, the primary behavioral and ERP group analyses were repeated after restricting the child group to participants aged at least 84 months. This yielded 18 children (M age = 7.83 years, *SD* = 0.77, range = 7.00–9.67) and retained all 67 adults. The behavioral model, planned contrasts, and Holm adjustments were identical to those used in the primary Group × Sentence Type analysis. The ERP sensitivity analysis likewise used the same posterior ROI, six prespecified 100-ms windows, model specification, directional within-group OSV > SOV tests, two-tailed adult–child interaction tests, and BH-FDR correction families as the primary ERP analyses.

**Supplementary Table S21.** Age-restricted behavioral sensitivity analysis including children aged 7 years or older.

**Panel A. Descriptive accuracy**

| **Group** | **n** | **Canonical SOV M (SD) [95% CI]** | **Noncanonical OSV M (SD) [95% CI]** | **SOV − OSV difference M (SD) [95% CI]** |
| --- | --- | --- | --- | --- |
| Adults | 67 | 96.64 (3.42) [95.81, 97.48] | 93.87 (7.50) [92.04, 95.70] | 2.77 (7.70) [0.89, 4.64] |
| Children ≥ 7 years | 18 | 76.06 (15.77) [68.22, 83.91] | 67.82 (15.89) [59.92, 75.72] | 8.24 (9.30) [3.62, 12.86] |

**Panel B. Omnibus tests**

| **Effect** | ***df*** | ***F*** | ***p*** |
| --- | --- | --- | --- |
| Group | 1, 83 | 124.08 | < .001 |
| Sentence type | 1, 83 | 26.52 | < .001 |
| Group × Sentence Type | 1, 83 | 6.56 | .012 |

**Panel C. Planned contrasts**

| **Contrast** | ***b*** | ***SE*** | **95% CI** | ***df*** | ***t*** | ***p*** |
| --- | --- | --- | --- | --- | --- | --- |
| Canonical SOV − Noncanonical OSV, adults | 2.77 | 0.98 | [0.81, 4.72] | 83 | 2.81 | .006 |
| Canonical SOV − Noncanonical OSV, children ≥ 7 | 8.24 | 1.90 | [4.47, 12.01] | 83 | 4.34 | < .001 |
| Child ≥ 7 SOV−OSV difference − adult SOV−OSV difference | 5.47 | 2.14 | [1.22, 9.72] | 83 | 2.56 | .012 |
| Adults − children ≥ 7, canonical SOV | 20.58 | 2.35 | [15.93, 25.23] | 123.52 | 8.76 | < .001 |
| Adults − children ≥ 7, noncanonical OSV | 26.05 | 2.35 | [21.40, 30.70] | 123.52 | 11.09 | < .001 |

*Note.* Accuracy values and coefficients are expressed in percentage points. The age-restricted child sample comprised 18 children aged 7.00–9.67 years. Panel B reports the same Group × Sentence Type model used for the primary behavioral analysis. In Panel C, Holm correction was applied separately across the two group-specific sentence-type contrasts and across the two condition-specific group contrasts; the child–adult difference in the SOV − OSV accuracy difference was a single planned contrast and was not additionally corrected.

**Supplementary Table S22.** Age-restricted ERP sensitivity analysis including children aged 7 years or older.

**Panel A. Directional noncanonical OSV − canonical SOV contrasts in children aged ≥ 7 years**

| **Time window (ms)** | ***b*** | ***SE*** | **95% CI** | ***t*(83)** | **Directional *p*** | **Directional *pFDR*** |
| --- | --- | --- | --- | --- | --- | --- |
| 600–700 | 0.749 | 0.673 | [−0.590, 2.088] | 1.11 | .134 | .268 |
| 700–800 | −0.029 | 0.476 | [−0.977, 0.918] | −0.06 | .525 | .629 |
| 800–900 | −0.289 | 0.462 | [−1.209, 0.631] | −0.63 | .733 | .733 |
| 900–1000 | 0.976 | 1.054 | [−1.119, 3.072] | 0.93 | .178 | .268 |
| 1000–1100 | 1.271 | 1.028 | [−0.774, 3.317] | 1.24 | .110 | .268 |
| 1100–1200 | 1.144 | 0.668 | [−0.186, 2.473] | 1.71 | .045 | .268 |

**Panel B. Adult–child differences in the OSV − SOV ERP effect**

| **Time window (ms)** | **Adult effect − child ≥ 7 effect, b** | ***SE*** | **95% CI** | ***t*(83)** | ***p*** | ***pFDR*** |
| --- | --- | --- | --- | --- | --- | --- |
| 600–700 | −0.227 | 0.694 | [−1.607, 1.153] | −0.33 | .744 | .744 |
| 700–800 | 0.408 | 0.504 | [−0.595, 1.411] | 0.81 | .421 | .631 |
| 800–900 | 0.668 | 0.498 | [−0.323, 1.658] | 1.34 | .184 | .631 |
| 900–1000 | −0.637 | 1.076 | [−2.777, 1.502] | −0.59 | .555 | .666 |
| 1000–1100 | −1.020 | 1.052 | [−3.111, 1.072] | −0.97 | .335 | .631 |
| 1100–1200 | −0.619 | 0.703 | [−2.016, 0.779] | −0.88 | .381 | .631 |

*Note.* Adults *n* = 67; children aged ≥ 7 years *n* = 18. Estimates are posterior ROI mean-amplitude differences in µV. Panel A reports the prespecified directional OSV > SOV tests within the age-restricted child sample; BH-FDR correction was applied across the six time windows. Panel B reports two-tailed adult–child comparisons of the OSV − SOV effect; positive estimates indicate a larger OSV − SOV effect in adults, and BH-FDR correction was applied across the six interaction contrasts. Confidence intervals are two-sided 95% intervals.

**References**

Aksan, Y., Aksan, M., Koltuksuz, A., Sezer, T., Mersinli, Ü., Demirhan, U. U., Yılmazer, H., Atasoy, G., Öz, S., Yıldız, İ., & Kurtoğlu, Ö. (2012). Construction of the Turkish National Corpus (TNC). In N. Calzolari, K. Choukri, T. Declerck, M. U. Doğan, B. Maegaard, J. Mariani, A. Moreno, J. Odijk, & S. Piperidis (Eds.), *Proceedings of the Eighth International Conference on Language Resources and Evaluation (LREC’12)* (pp. 3223–3227). European Language Resources Association. https://aclanthology.org/L12-1590/

Eskioğlu, E. İ., Günhan Şenol, N. E., & Bulut, T. (2025). Action and object picture naming in Turkish: A psycholinguistic database. *Behavior Research Methods, 57*(8), Article 225. https://doi.org/10.3758/s13428-025-02764-6

Kuperman, V., Stadthagen-Gonzalez, H., & Brysbaert, M. (2012). Age-of-acquisition ratings for 30,000 English words. *Behavior Research Methods, 44*(4), 978–990. https://doi.org/10.3758/s13428-012-0210-4
